# Bevacizumab’s Association With a Decreased Risk of Brain Metastases in ECOG-ACRIN E1505, a Phase 3 Randomized Trial of Adjuvant Chemotherapy With or Without Bevacizumab in Surgically Resected NSCLC

**DOI:** 10.1016/j.jtocrr.2021.100274

**Published:** 2022-01-10

**Authors:** John M. Varlotto, Yating Wang, Zhuoxin Sun, Heather A. Wakelee, Suresh Ramalingam, Joan Schiller

**Affiliations:** a Department of Oncology, Marshall University, Huntington, West Virginia; b Department of Biostatistics & Computational Biology, Dana-Farber Cancer Institute, Boston, Massachusetts; c Division of Oncology, Department of Medicine, Stanford University School of Medicine, Stanford, California; d Department of Hematology and Medical Oncology, Winship Cancer Institute, Emory University School of Medicine, Atlanta, Georgia; e Department of Medical Oncology, Inova Schar Cancer Institute, Falls Church, Virginia

**Keywords:** Brain metastases, Non–small cell lung cancer, Adjuvant therapy, Bevacizumab

## Abstract

**Introduction:**

ECOG-ACRIN E1505 was a phase 3 randomized trial of adjuvant chemotherapy with or without bevacizumab for patients with stages IB (>4 cm) to IIIA NSCLC. We sought to estimate the incidence and risk factors for brain recurrence as compared with extracranial recurrences (ECRs).

**Methods:**

ECOG-ACRIN E1505 noted that bevacizumab failed to improve overall survival (OS) (OS hazard ratio [HR] = 0.99 [0·82–1·19], *p* = 0.90) or recurrence-free survival when added to chemotherapy in the adjuvant setting. The cumulative incidence of brain/ECR was estimated after adjusting for recurrence at other sites and death as competing events. A multivariable regression model was fitted using competing risk analysis to evaluate the effect of covariates on brain recurrence incidence.

**Results:**

Median follow-up was 50.4 months. Among the 1501 patients enrolled, 472 developed ECR. There were 122 patients who had recurrence in the brain with or without simultaneous ECR as the first recurrence site (all-brain recurrences [ABRs]), and 84 of those with ABRs had recurrence in the brain only (isolated-brain recurrence [IBR]). The incidence of ABR, IBR, and ECR at 6 years was 9.9%, 5.9%, and 38.8%, respectively. Chemotherapy plus bevacizumab was associated with a decreased incidence of ABR (HR = 0.64, *p* = 0.02) and IBR (HR = 0.62, *p* = 0.032), but there was no significant trend for an OS decrement in the bevacizumab arm versus the control arm for both ABR and IBR. Median survivals associated with IBR, ABR, and ECR were 9.5, 9.5, and 14.1 months, respectively. Nonsquamous histology (HR = 1.87, *p* = 0.003) was also associated with ABR. ECR was associated with nonsquamous NSCLC histology (HR = 1.79, *p* < 0.01) and stage/N2 involvement (HR = 1.13/1.37, both *p* < 0.01).

**Conclusions:**

The addition of bevacizumab to chemotherapy was associated with reduction in brain recurrences, but not ECR. Brain metastases whether isolated or not are associated with a lower median survival than ECR and unlike ECR are not associated with traditional staging variables.

## Introduction

Past retrospective series have revealed that the incidence of brain metastases after surgical resection of early stage NSCLC approaches 10% by 5 years.^1^^,^^2^ Nevertheless, those series contained many patients who were treated in the era before the establishment of effective adjuvant chemotherapy.^3^, ^4^, ^5^ The incidence of brain metastases and their associated risk factors have not been well-described when optimal surgical therapy and adjuvant chemotherapy were uniformly given. ECOG/ACRIN E1505 was a randomized phase 3 trial that investigated the potential overall survival (OS) benefit of bevacizumab in addition to standard chemotherapy in completely resected, early stage NSCLC.^6^ Bevacizumab was not associated with a survival benefit.^6^ The purpose of our report is to use the failure pattern data from ECOG-ACRIN E1505 to further define the cumulative incidence of all-brain recurrences (ABRs) and isolated-brain recurrence (IBR) as a site of first failure, and the treatment, patient, or histologic variables associated with brain metastases as compared with extracranial recurrences (ECRs). In addition, we wanted to evaluate whether brain recurrences are associated with a shorter survival as compared with ECRs.

## Material and Methods

### Patient Eligibility and Treatment

ECOG-ACRIN E1505 was a phase 3 randomized trial that involved 1501 patients with early stage NSCLC (stages IB [≥4 cm], II, and IIIA), squamous NSCLC (SQ-NSCLC), and non–SQ-NSCLC (NS-NSCLC) tumors who underwent complete resection (R0) by pneumonectomy or lobectomy. Sublobar resections were not permitted. Mediastinal lymph node sampling was required preoperatively by mediastinoscopy or intraoperatively (levels 7 and 4R for right-sided tumors or levels 7, 5, and 6 for left-sided tumors). No brain imaging was mandated per protocol, though a brain magnetic resonance imaging was “strongly encouraged” for all patients with stage IIIA disease. Eligible patients were randomized to receive adjuvant chemotherapy or chemotherapy plus bevacizumab. Chemotherapy consisted of cisplatin doublets with an investigator’s choice of vinorelbine, docetaxel, gemcitabine, or pemetrexed. Patients were stratified by type of chemotherapy, American Joint Committee on Cancer sixth stage, histology (SQ cell versus other), and sex. The primary study end point was to evaluate whether bevacizumab in addition to standard adjuvant chemotherapy increased survival^6^ as previously found in advanced NSCLC.^7^ All-brain and ECRs as mentioned in this manuscript include only the first site of failure. Secondary recurrences were not included in our analysis.

### Follow-Up

Full details of the follow-up of patients have been previously noted.^6^ Patients were seen at 6-week intervals during the initial 3 months post-treatment. Chest radiograph was performed every 3 months during the first 2 years after registration, then every 6 months during years 2 to 5, and yearly afterward until years from the date of registration. All patients including those who terminated the protocol therapy early were followed for response until recurrence and for survival for 10 years from registration. Chest computed tomography (CT) scans were permitted as substitution for chest radiograph. Disease recurrences were encouraged to be documented by biopsy and to be fully restaged including a CT scan of the thorax and abdomen, brain imaging (preferably magnetic resonance imaging, but CT was acceptable), and a radionuclide bone scan or positron emission tomography scan. Brain imaging was not required in the follow-up and was generally obtained when patients became symptomatic or had recurrence elsewhere. Date of randomization to the date of first treatment failure (recurrence or death before recurrence) was recorded.

### Statistical Methods

The cumulative incidences of ABR, IBR, and ECR were estimated after adjusting for recurrence at other sites and death as competing events. ABRs referred to all brain recurrences whether occurring in isolation (IBR) or associated with extracranial failure. ECRs are all recurrences not involving the brain. Univariate analysis was performed to determine factors associated with the development of first recurrence sites. When comparing variables in Table 1 in the univariate analyses, Wilcoxon tests^8^ were used to compare continuous variables, Fisher’s exact test^9^ was used for categorical variables with two categories, and chi-square testing^9^ was used for categorical variables with more than two categories. The variables with *p* value less than 0.15 in the univariate analyses were selected for the multivariate analyses. A multivariable regression model was fitted using the methodology of Fine and Gray^10^ to evaluate the effect of covariates on recurrence adjusting for the competing. The following variables were analyzed to evaluate their association with brain metastases as a first site of failure: age, sex, race, ethnicity, chemotherapeutic regimen, tumor histology, bevacizumab, stage, weight loss, pathologic node stage, weight loss, performance status, tumor location, tumor size, resection type, lymph node dissection type (complete, sampling), co-morbidities (cardiovascular disease, hypertension, thrombotic events, myocardial infarction, [un]stable angina, other cardiovascular diseases), high baseline blood pressure, smoking history, percentage of positive N1/N2 nodes, and antihypertensive drugs.

**Table 1:** Demographic Factors for Total Population and the Three Recurrent Populations

Variables		Total Patients	ABR	*p*^a^	ECR	*p*^b^	IBR	*p*^c^
Patient#		1501	122	—	472	—	84	—
Age	Mean (SD)	60.8 (8.8)	59.8 (8.4)	0.168	60.9 (8.7)	0.801	60.1 (8.0)	0.513
	Median (Q1, Q3)	61 (55, 67)	60 (53, 66)		61 (55, 67)		62 (56, 66)	
	[min, max]	[30, 86]	[40, 79]		[37, 84]		[40, 79]	
	Freq. of missing	0	0		0		0	
Sex	Male	746 (50)	54 (44)	0.22	227 (48)	0.405	40 (48)	0.737
	Female	755 (50)	68 (56)		245 (52)		44 (52)	
	Unknown/missing	0	0		0		0	
Race	White	1302 (88)	110 (90)	0.814	403 (86)	0.374	77 (92)	0.538
	Black	131 (9)	10 (8)		50 (11)		7 (8)	
	Asian	38 (3)	2 (2)		11 (2)		0 (0)	
	Native Hawaiian	5 (0)	0 (0)		1 (0)		0 (0)	
	Native American	6 (0)	0 (0)		3 (1)		0 (0)	
	Unknown/missing	19	0		4		0	
Ethnicity	Hispanic	48 (3)	1 (1)	0.173	12 (3)	0.347	1 (1)	0.516
	Non-Hispanic	1368 (97)	113 (99)		437 (97)		78 (99)	
	Unknown/missing	85	8		23		5	
Chemotherapy	Cis/vinorelbine	377 (25)	37 (30)	0.543	111 (24)	0.562	22 (26)	0.404
	Cis/docetaxel	343 (23)	28 (23)		103 (22)		23 (27)	
	Cis/gemcitabine	283 (19)	21 (17)		92 (19)		18 (21)	
	Cis/pemetrexed	497 (33)	36 (30)		166 (35)		21 (25)	
	Unknown/missing	1	0		0		0	
Histology	Squamous	422 (28)	22 (18)	0.03	95 (20)	<0.01	17 (20)	0.148
	Adenocarcinoma	874 (58)	80 (66)		309 (65)		54 (64)	
	Large cell	38 (3)	6 (5)		10 (2)		4 (5)	
	BAC	13 (1)	0 (0)		5 (1)		0 (0)	
	NOS	40 (3)	6 (5)		14 (3)		5 (6)	
	Combined/mixed	93 (6)	8 (7)		29 (6)		4 (5)	
	Other	20 (1)	0 (0)		10 (2)		0 (0)	
	Unknown/missing	1	0		0		0	
Stage (by sx eval)	IB T2N0	383 (26)	28 (23)	0.296	83 (18)	<0.01	22 (27)	0.66
	IIA T1N1	174 (12)	12 (10)		43 (9)		7 (9)	
	IIB T2N1	394 (27)	40 (33)		126 (27)		28 (34)	
	IIB T3N0	68 (5)	4 (3)		19 (4)		3 (4)	
	IIIA T1N2	115 (8)	9 (8)		42 (9)		5 (6)	
	IIIA T2N2	243 (17)	25 (21)		116 (25)		15 (18)	
	IIIA T3N2	20 (1)	1 (1)		7 (2)		1 (1)	
	IIIA T3N1	61 (4)	1 (1)		23 (5)		1 (1)	
	Unknown/missing	43	2		13		2	
Weight loss	<5%	1186 (79)	99 (81)	0.95	380 (81)	0.604	67 (80)	0.974
	5% to <10%	209 (14)	15 (12)		62 (13)		11 (13)	
	10% to <20%	91 (6)	7 (6)		27 (6)		5 (6)	
	>20%	12 (1)	1 (1)		2 (0)		1 (1)	
	Unknown/missing	3	0		1		0	
PS	Fully active	879 (59)	70 (57)	0.774	276 (58)	0.955	45 (54)	0.362
	Ambulatory	620 (41)	52 (43)		196 (42)		39 (46)	
	Unknown/missing	2	0		0		0	
Pathologic node stages	PN0	441 (32)	28 (27)	0.411	93 (22)	<0.01	22 (31)	0.91
	PN1	589 (43)	46 (44)		176 (42)		32 (45)	
	PN2	351 (25)	31 (30)		150 (36)		17 (24)	
	Unknown/missing	120	17		53		13	
Tumor size	Mean (SD)	4.67 (2.66)	4.82 (2.45)	0.293	4.85 (3.03)	0.953	4.97 (2.29)	0.078
	Median (Q1, Q3)	4.2 (2.8,6.0)	4.5 (3.0,6.5)		4.1 (2.7,6.0)		4.7 (3.2,6.1)	
	[min, max]	[0.4,28.0]	[0.8,13.0]		[0.8,24.0]		[0.8,11.0]	
	Freq. of missing	2	0		0		0	
Resection type	Intraperi. pneumonectomy	8 (1)	1 (1)	0.245	4 (1)	0.382	1 (1)	0.586
	Pneumonectomy	184 (12)	22 (18)		58 (12)		15 (18)	
	Lobectomy	1134 (76)	88 (72)		355 (75)		60 (71)	
	Bilobectomy	105 (7)	5 (4)		33 (7)		4 (5)	
	Sleeve lobectomy	22 (1)	3 (2)		3 (1)		2 (2)	
	Lobectomy and chest wall resect	31 (2)	1 (1)		13 (3)		1 (1)	
	Other	16 (1)	2 (2)		6 (1)		1 (1)	
	Unknown/missing	1	0		0		0	
LN dissection type	None	1 (0)	0 (0)	0.325	0 (0)	0.316	0 (0)	0.35
	Incomplete sampling	89 (6)	12 (10)		20 (4)		7 (8)	
	Systematic sampling	689 (46)	58 (48)		227 (48)		44 (52)	
		701 (47)	50 (41)		220 (47)		31 (37)	
	Other	19 (1)	2 (2)		5 (1)		2 (2)	
	Unknown/missing	2	0		0		0	
Smoke after diagnosis	Yes	467 (40)	43 (47)		136 (37)		33 (49)	
	Refused to answer	5 (0)	0 (0)		2 (1)		0 (0)	
	Unknown/missing	335	30		106		17	
Currently smoking	No	1176 (88)	94 (85)	0.361	368 (90)	0.17	67 (85)	0.371
	Yes	160 (12)	16 (15)		41 (10)		12 (15)	
	Unknown/missing	165	12		63		5	
Cigarettes per day	Mean (SD)	24.0 (12.8)	24.2 (10.8)	0.581	23.2 (12.9)	0.027	25.2 (10.8)	0.167
	Median (Q1, Q3)	20 (20, 30)	20 (20, 30)		20 (15, 30)		20 (20, 30)	
	[min, max]	[0, 100]	[1, 60]		[0, 80]		[1, 60]	
	Freq. of missing	181	15		77		5	
Bevacizumab	Without bevacizumab	749 (50)	74 (61)	0.014	254 (50)	1	52 (62)	0.025
	With bevacizumab	752 (50)	48 (39)		256 (50)		32 (38)	
	Unknown/missing	0	0		0		0	
Percentage of N1 positive	Mean (SD)	0.1 (0.2)	0.2 (0.3)	0.363	0.2 (0.3)	<0.01	0.1 (0.3)	0.64
	Median (Q1, Q3)	0 (0, 0)	0 (0, 0)		0 (0, 0)		0 (0, 0)	
	[min, max]	[0, 1]	[0, 1]		[0, 1]		[0, 1]	
	Freq. of missing	53	7		21		4	
Percentage of N2 positive	Mean (SD)	0.4 (0.4)	0.5 (0.4)	0.192	0.5 (0.4)	<0.01	0.4 (0.4)	0.614
	Median (Q1, Q3)	0 (0, 1)	0 (0, 1)		0 (0, 1)		0 (0, 1)	
	[min, max]	[0, 1]	[0, 1]		[0, 1]		[0, 1]	
	Freq. of missing	145	12		46		8	

#, number; ABR, all-brain recurrence; BAC, bronchioloalveolar carcinoma; ECR, extracranial recurrence; Freq., frequency; IBR, isolated brain recurrence; intraperi., intraperitoneal; max, maximum; mets; metastases; min, minimum; LN, lymph node; NOS, not otherwise specified; PS, performance status; Q, quartile.

p-values were reflect the difference between that recurrence type and the population without the specific recurrence.

a *p* values to compare patients with brain mets versus without brain mets on the basis of univariate analysis.

b *p* values to compare patients with extracranial recurrence versus without extracranial recurrence on the basis of univariate analysis.

c *p* values to compare patients with isolated brain mets versus without isolated brain mets on the basis of univariate analysis.

To evaluate the differences of OS after recurrences between bevacizumab and control arms, multivariate Cox models were fitted including time to recurrence as covariate. All *p* values were based on two-sided tests.

## Results

With a median follow-up of 50.4 months, a total of 122 patients developed brain metastases (74 in control arm and 48 in bevacizumab arm) as the first site of recurrence with or without other simultaneous sites of recurrence (ABR). The incidence of ABR at 1-year postrandomization was 3.8% (95% confidence interval [CI]: 2.8%–4.8%), and it increased to 8.6% (95% CI: 7.0%–10.1%) at 3 years and to 9.9% (95% CI: 8.1%–11.7%) at 6 years. The incidence of ABR at 1 year, 3 years, and 6 years postrandomization in the control/bevacizumab arms was 5.4% (95% CI: 3.8%–7.1%)/2.2% (95% CI: 1.1%–3.2%), 10.6% (95% CI: 8.3%–13.0%)/6.4% (95% CI: 4.6%–8.3%), and 11.6% (95% CI: 8.8%–14.4%)/8.2% (95% CI: 5.8%–10.5%), respectively.

**Figure 1:**
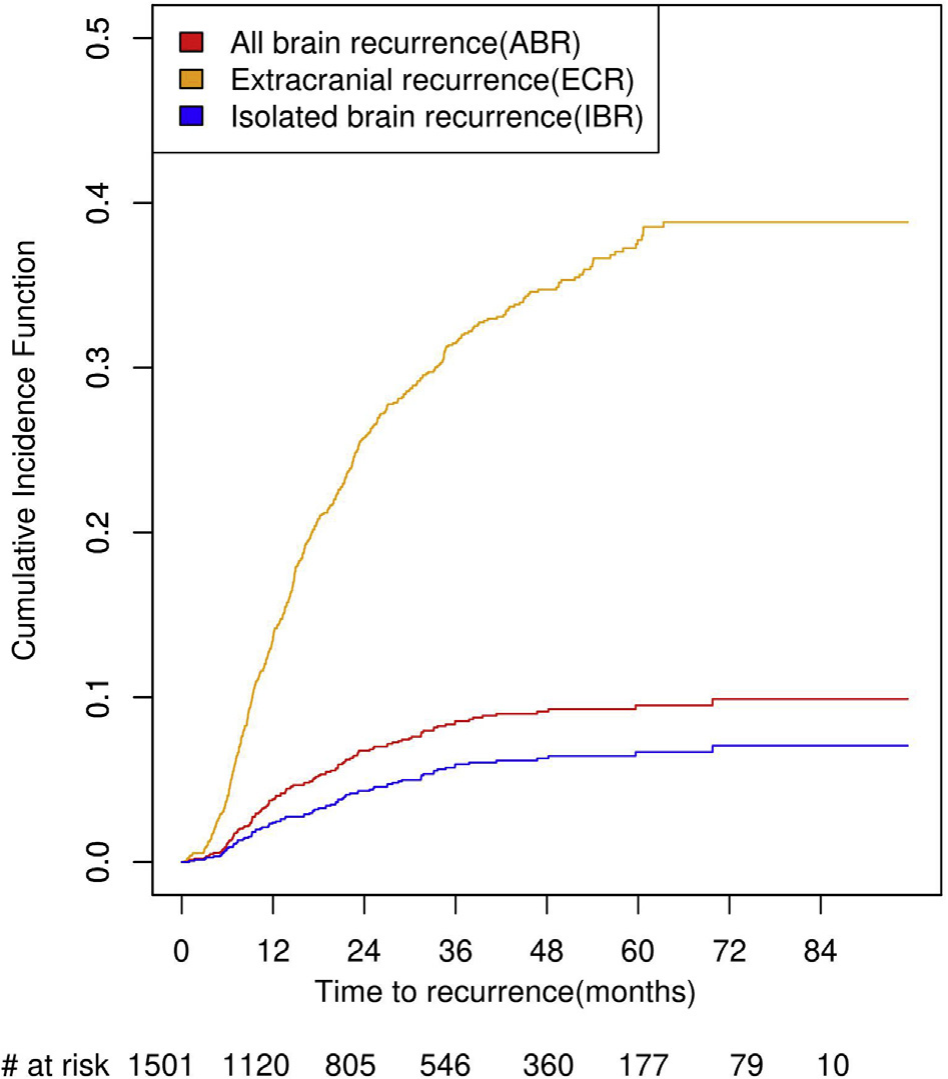
Cumulative incidence of ABR, IBR, and ECR versus time in the overall population. ABR, all-brain recurrence; ECR, extracranial recurrence; IBR, isolated-brain recurrence; No. Number.

**Figure 2:**
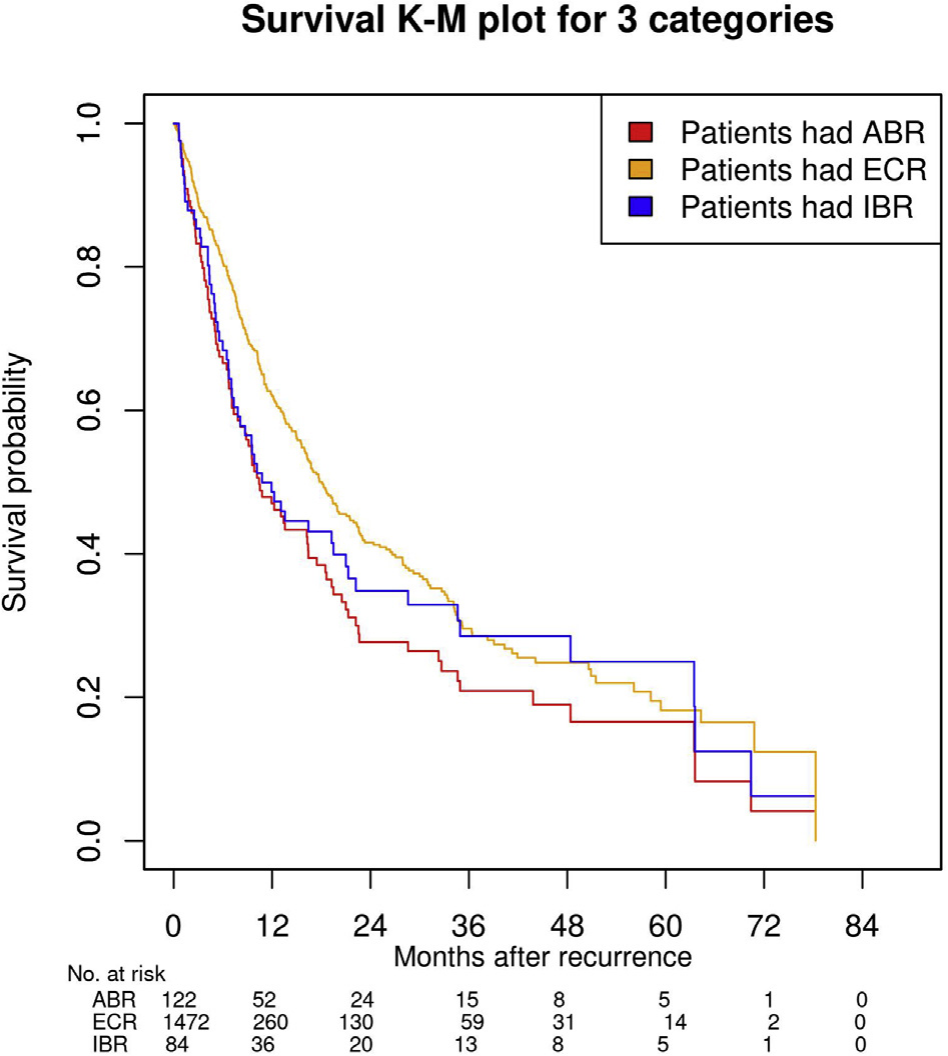
Survival after ABR, IBR, and ECR versus time in the overall population. ABR, all-brain recurrence; ECR, extracranial recurrence; IBR, isolated-brain recurrence; K-M, Kaplan-Meier; No. Number.

A total of 84 patients (52 in control arm, 32 in bevacizumab arm) had isolated brain metastases as the first site of recurrence (IBR). The incidence of IBR at 1 year, 3 years, and 6 years was 2.4% (95% CI: 1.6%–3.2%), 5.9% (95% CI: 4.6%–7.2%), and 7.1% (95% CI: 5.4%–8.7%), respectively. The incidence of IBR at 1 year, 3 years, and 6 years for the control/bevacizumab arms was 3.6% (95% CI: 2.3%–5.0%)/1.1% (95% CI: 0.3%–1.9%), 7.7% (95% CI: 5.6%–9.7%)/4.2% (95% CI: 2.6%–5.7%), and 8.4% (95% CI: 5.9%–11.0%)/5.7% (95% CI: 3.6%–7.7%), respectively. A total of 472 patients (232 in control arm, 240 in bevacizumab arm) had extracranial metastases (ECRs) as the first site of recurrence. The incidence of ECR at 1 year, 3 years, and 6 years was 13.7% (95% CI: 11.9%–15.5%), 31.5% (95% CI: 29.0%–34.0%), and 38.8% (95% CI: 35.8%–41.9%), respectively. The incidence of ECR at 1 year, 3 years, and 6 years in the control/bevacizumab arms was 14.8% (95% CI: 12.2%–17.4%)/12.6% (95% CI: 10.1%–15.0%), 30.1% (95% CI: 26.7%–33.6%)/32.9% (95% CI: 29.3%–36.6%), and 37.0% (95% CI: 32.8%–41.2%)/40.8% (95% CI: 36.3%–45.2%), respectively. The three recurrence patterns can be found in Figure 1. The median survival (mos) after experiencing these recurrences is as follows: 9.51 (ABR), 9.53 (IBR), and 14.1(ECR) for the entire population. Figure 2 graphs the survival for these three recurrence types. Median survival in the bevacizumab and control arms for patients with ABR was 6.9 and 10.3 months (HR = 1.46, 95% CI: 0.94–2.26, *p* = 0.090); for IBR, 5.8 and 10.5 months (HR = 1.50, 95% CI: 0.86–2.62, *p* = 0.151); and for ECR, 14.5 and 13.6 months (HR = 1.02, 95% CI: 0.81–1.29, *p* = 0.845), respectively.

To search whether the trends for worse survival for ABR and IBR were due to bevacizumab masking symptoms and contrast enhancement, the survival of the ABR patient group was split into a period before and after 18 months. In the 35 patients who developed brain metastases before or at 18 months postrandomization, 11 patients were in the bevacizumab arm and 24 patients were in the control arm and had median survivals after recurrence of 2.73 months and 3.88 months, respectively (HR = 1.26 and *p* = 0.613). In the 87 patients who developed brain metastases after 18 months postrandomization, 37 patients were in the bevacizumab arm and 50 were in the control arm with median survival after recurrence of 12.09 months and 18.53 months, respectively (HR = 1.52 and *p* = 0.133).

Demographic factors associated with patient characteristics, histopathology, and treatment can be found in Table 1 (Supplementary Table 1 for all variables analyzed) for all three recurrence populations. The tables compare all three recurrence populations to the population without that particular recurrence. Patients with ABR were less likely to have SQ histology (*p* = 0.03) and less likely to have received bevacizumab (*p* = 0.014). Patients with ECR were also less likely to have SQ histology (*p* < 0.01) but were more likely to have a higher tumor (*p* < 0.01)/nodal stage (*p* < 0.01), heavier cigarette use (*p* = 0.062), and a greater percentage of N1 (*p* < 0.01)/N2(*p* < 0.01) nodal positivity. The only factor significant for IBR was chemotherapy without bevacizumab (*p* < 0.025).

**Figure 3:**
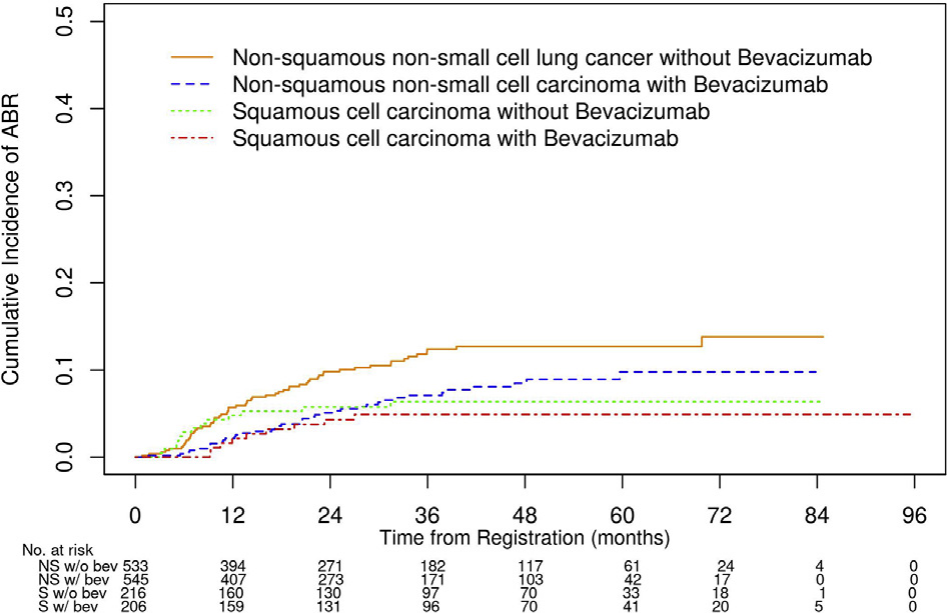
The probability of developing ABRs in the SQ-NSCLC and NS-NSCLC groups by whether or not bev was given. ABR, all-brain recurrence; bev, bevacizumab; NS, nonsquamous; SQ, squamous; w/, with; w/o, without.

Univariate analysis of the variables to evaluate their association with ABR as a first site of recurrence can be found in Supplementary Table 2. The two factors most associated with a risk of ABR are histology (*p* = 0.03) and use of bevacizumab (*p* = 0.014). Multivariate analysis of factors associated with ABR noted that both tested factors were significant, including NS histology (HR = 1.87, 95% CI: 1.171–2.98, *p* = 0.0087) and bevacizumab (HR = 0.64, 95% CI: 0.45–0.92, *p* = 0.0170). In patients who did not receive bevacizumab, the probability of having brain metastases at 1 year, 3 years, and 6 years was 4.79%, 6.37%, and 6.37% in the SQ group and 5.71%, 12.39%, and 13.8% in the NS group, respectively (Fig. 3 and Supplementary Table 3). In the patients who received bevacizumab, the probability of having brain metastases at 1 year, 3 years, and 6 years was 2.14%, 4.88%, and 4.88% in the SQ group and 2.16%, 7.09%, and 9.78% in the NS group, respectively (Fig. 3).

**Figure 4:**
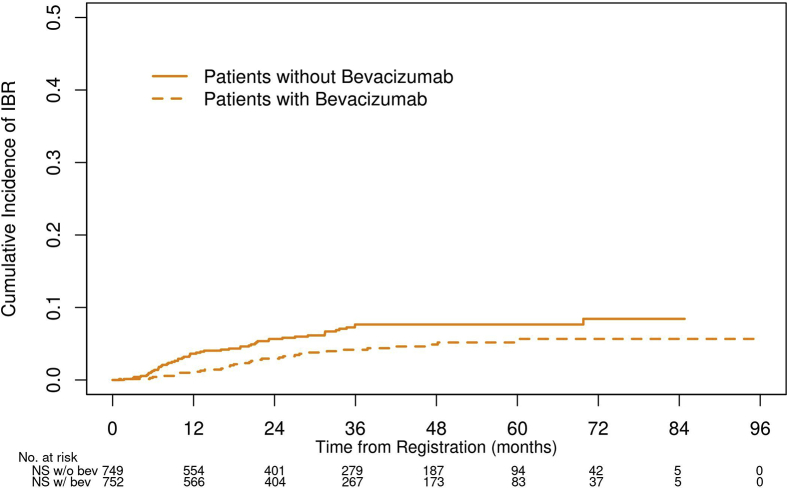
IBRs by administration of bev. IBR, isolated-brain recurrence; bev, bevacizumab; NS, nonsquamous; w/, with; w/o, without.

Univariate analysis of the variables to evaluate their association with IBR as a first site of recurrence can be found in Supplementary Table 4. The three factors most associated with a risk of IBR are histology (*p* = 0.148), tumor size (*p* = 0.078), and bevacizumab (*p* = 0.025). Although NS-NSCLC histology (HR = 1.68, 95% CI: 0.98–2.87, *p* = 0.058) and tumor size (HR = 1.05, 95% CI: 1.00–1.10, *p* = 0.076) were not significantly associated with IBR in the multivariate analysis, bevacizumab was associated with a lower risk of brain metastases (HR = 0.62, 95% CI: 0.40–0.96, *p* = 0.032, Fig. 4).

Univariate analysis of the variables to evaluate their association with ECR as a first site of recurrence can be found in Supplementary Table 5. The factors associated with ECR included histology (*p* < 0.01), stage (*p* < 0.01), pNodal stage (*p* < 0.01), cigarettes/d (*p* = 0.06), and %N1 (*p* < 0.01)/%N2 (*p* < 0.01) nodes positive. Multivariate analysis was not significant for cigarettes/d (HR = 1.00, 95% CI: 0.99–1.01, *p* = 0.27), pNodal stage (HR = 1.10, 95% CI: 0.83–1.45, *p* = 0.46), and %N1 nodes positive (HR = 1.26, 95% CI: 0.69–2.28, *p* = 0.45), but the analysis was significant for NS-NSCLC histology (HR = 1.79, 95% CI: 1.40–2.43, *p* < 0.01), stage (HR = 1.13, 95% CI: 1.05–1.22, *p* < 0.01), and %N2 positive (HR = 1.37, 95% CI: 1.03–1.82, *p* < 0.01).

The CNS was the second most common site of isolated metastasis at 19% of isolated recurrences as compared with the liver (2%), lung (43%), skeletal (9%), nodal (14%), and others (13%). In those who had recurrence at more than one site or isolated sites, the CNS was involved in 15.4% of the recurrences as compared with the liver (5%), lung (36.4%), skeletal (14.2%), nodal (15.4%), other (13.5%), and unknown (0.3%).

## Discussion

Surgical resection with curative intent remains the established standard for patients with clinically operable, early stage NSCLC. Since 2010, more than 82,000 pneumonectomies or lobectomies were performed annually in the United States.^11^ Because brain recurrences occurred in approximately 10% of the surgical population, the development of brain recurrence is a serious problem because it can affect more than 8000 patients yearly in the United States. Nevertheless, it is hoped that the number of brain recurrences will be decreased by finding earlier stages of lung cancer by CT scan screening.^12^

There have been many studies evaluating the incidence and risk factors associated with brain metastases in patients with lung cancer who are treated definitively. We have listed those studies that contained at least 25 patients who developed brain metastases in three tables (Supplementary Tables 6–8). These tables reveal those studies investigating brain metastases that developed at any time after definitive treatment or those brain metastases as a site of isolated/first site of recurrence^1^^,^^2^^,^^13^, ^14^, ^15^, ^16^, ^17^, ^18^, ^19^, ^20^ and those studies with the development of brain metastases in prospective, randomized trials of prophylactic whole brain radiation therapy (WBRT) (Supplementary Table 8).^21^, ^22^, ^23^, ^24^, ^25^, ^26^ Supplementary Table 8 contains information relevant to only the patients in the observation arm of the prospective, randomized trials investigating the role of WBRT so that the risk factors and incidence may be more comparable with our patient population who had also undergone a similar rigorous follow-up in a phase 3 trial. The current series contains the largest “at risk” group for the development of brain metastases, and it also has the benefit of prospective follow-up, albeit with a post hoc analysis. Excluding two retrospective studies^1^^,^^2^ which analyzed only those patients undergoing surgical resection, all other retrospective series dealt with predominantly more advanced stages that were generally treated with multimodality therapy and had calculated rates of brain metastases at 24.2% to 39.8% at 2 to 3 years,^13^^,^^14^^,^^17^^,^^18^^,^^20^ which are much higher than the rates in our patient population.

The prospective, randomized trials generally dealt with patients treated by definitive radiation and reported crude rates of brain metastases of 13% to 27%, revealing although prophylactic WBRT can decrease the incidence of brain metastases, there was no OS benefit associated with radiation.^21^, ^22^, ^23^, ^24^ Nevertheless, it should be noted that the study reported from Li et al.^25^ was terminated early because the survival benefit was not significant despite the primary end point of an increased disease-free survival benefit with WBRT being realized.

Despite the differences in patient populations and methodology, our results are in alignment with most past series because they also revealed that NS and adenocarcinoma histologies were associated with the development of brain metastases.^1^^,^^2^^,^^13^, ^14^, ^15^, ^16^^,^^18^^,^^19^^,^^21^ Although our patient population did not have any association of brain metastases with patient age or node involvement, several series noted that younger patients^1^^,^^13^^,^^21^^,^^25^ and advanced node involvement/stage^1^^,^^14^, ^15^, ^16^^,^^18^ were associated with an increased risk of brain metastases. Interestingly, traditional prognostic factors (stage and % of N2 nodes positive) were associated with ECR, not either form of brain recurrence (IBR or ABR) in our investigation.

It should be noted that bevacizumab was the only factor associated with IBR by multivariate analysis and was also found to be associated with ABR by means of multivariate analysis and by Fisher’s exact test (Table 1) for the IBR and ABR populations. We found this association between bevacizumab and the prevention of brain metastases to be surprising because the addition of bevacizumab to chemotherapy did not increase the survival over that of patients receiving chemotherapy alone in ECOG-ACRIN E1505 and because the development of brain metastases, either IBR or ABR, was associated with shorter median survivals (both 9.5mo) than that of patients who had only ECR (14.1 mo).

The role of bevacizumab in preventing or delaying brain metastases has been evaluated in patients with metastatic disease, but not earlier stages of lung cancer until our analysis. An investigative group evaluated whether bevacizumab was associated with a reduction in brain metastases by retrospectively analyzing data from patients with metastatic cancer who were treated on prospective, randomized trials of chemotherapy with or without bevacizumab for breast cancer (AVADO and AVAREL trials) and for NS-NSCLC (the AVAiL trial).^27^ Although bevacizumab had no effect on the development of brain metastases in the breast cancer studies, the retrospective evaluation of the AVAiL trial revealed that bevacizumab was associated with a reduction in brain metastases as a site of first recurrence compared with chemotherapy alone (2.6% versus 5.8%). Furthermore, they found that patients receiving bevacizumab had a prolonged time to the development of brain metastases (7.8 versus 4.5 mo). These same authors reported that brain metastases could be prevented in mice with the use of subclinical doses of bevacizumab^27^ and hypothesized that low-dose bevacizumab may be useful for this purpose in patients. Post hoc analysis of the IMpower150 study^28^ which randomized patients to three arms (ABCP [bevacizumab/atezolizumab/chemotherapy], BCP [bevacizumab/chemotherapy], and ACP [atezolizumab/chemotherapy]) noted that there were higher rates of brain metastases in the arm without bevacizumab (ACP, 11.9%) as compared with the arms with bevacizumab (ABCP 7.0% and BCP 6.0%). This study noted that the time to development of brain metastases was delayed in the ABCP arm as compared with the BCP arm (HR = 0.68, 95% CI: 0.39–1.19), suggesting immunotherapy may act synergistically with bevacizumab to delay the development of brain metastases. Bevacizumab was also found in a small retrospective study to reduce the incidence of brain metastases when given with chemotherapy as compared with chemotherapy alone.^29^ When we looked at the median survivals for the three types of recurrence associated within the different treatment arms of ECOG-ACRIN E1505, we noticed an interesting finding that the median survivals of ABR and IBR were numerically, but not significantly lower in the bevacizumab arm than in the control arm (6.9 versus 10.3 mo for ABR and 5.8 versus 10.5 mo). The survival differences for ECR were much less pronounced and noted to be slightly higher, but not significantly in the bevacizumab arm as compared with the control arm (14.5 mo and 13.6 mo). We speculated that the lower survivals associated with both ABR and IBR in the bevacizumab arm may have been due to a delay of symptoms in this arm because bevacizumab may have delayed diagnosis because of its well-known ability to treat intracranial symptoms.^30^ Furthermore, even if brain imaging was obtained, bevacizumab may have delayed diagnosis by preventing the metastatic lesions from enhancing.^31^ Therefore, we evaluated those who had developed brain metastases less than or equal to 18 months and afterward with the thought that the period of 18 months would account for the administration and long half-life of bevacizumab (21 d). Nevertheless, we found that the OS of patients treated with bevacizumab was shorter in both time periods, 2.73 versus 3.88 months and 12.09 versus 18.53 months, respectively. This analysis revealed the virulence of metastases occurring in the early time period after surgery and that the survival of brain metastases in the bevacizumab arm was worse whether occurring early or late after surgery.

Although our analysis is the largest study to our knowledge that evaluates an at-risk, definitively treated lung cancer population for the development of brain metastases and uses patient data from a prospective trial, our analysis is post hoc and retrospective and is subject to bias. Brain imaging was not required before randomization in this trial, and thus, some patients may have had asymptomatic, undiagnosed brain metastases at the time of entry onto the study. Because there was no active screening for brain metastases, our incidence of brain metastases of 9.9% at 6 years may be an underestimation particularly as patients may have died of unknown causes or had otherwise undetected brain metastases when they presented with symptomatic widely metastatic extracranial disease. Furthermore, our database was missing many factors, including lymphatic vascular invasion, perineural invasion, transfusions, and the updated adenocarcinoma pathologic categorization.^32^ Nevertheless, our study is the first to note that bevacizumab may prevent brain metastases in a lower risk population undergoing definitive therapy.

Because brain metastasis as either IBR or ABR was the second most common site of metastases in both situations (isolated organ involvement or with other metastatic lesions) and because the development of brain metastases was associated with a lower median survival (9.5 months for both IBR and ABR) as compared with ECRs (14.1 mo), the prevention of brain metastases in the postsurgical setting by a new therapeutic approach or by finding a high-risk population for surveillance would be very beneficial to patient outcomes. Perhaps, in the future, we will have molecular markers for the prediction of brain metastases beyond the currently known driver mutations.

We feel that our results may help to spur prospective trials in the adjuvant setting. Currently, we cannot recommend bevacizumab or other antiangiogenic agents adjuvantly for a high-risk patient population at risk for brain metastases because there is no OS benefit associated with this agent,^6^ there was no survival benefit in the patients with brain metastases associated with bevacizumab in this investigation, and bevacizumab has toxicities in addition to adjuvant chemotherapy.^6^ Immune checkpoint inhibitors have been found to cross the blood brain barrier. Recently, prospective trials in patients with untreated NSCLC with brain metastases have noted that pembrolizumab results in a 29.7% response rate in patients with tumors with a programmed death-ligand 1 (PD-L1) of greater than or equal to 1%^33^ and that the combination of atezolizumab, carboplatin, and pemetrexed for NS-NSCLC resulted in a median intracranial progression-free survival of 6.9 months, an intracranial progression-free survival of 10.4% at 18 months, and similar objective response rates intracranially and extracranially of 40.0% and 47.5%, respectively.^34^ On October 15, 2021, the Food and Drug Administration approved atezolizumab for the adjuvant treatment of NSCLC after resection and platinum-based chemotherapy for patients whose tumor expressed PD-L1 of 1% or greater.^35^ Nevertheless, the benefit was largely confined to the patient group with tumors expressing PD-L1 of greater than or equal to 50%.^36^ Of interest, antiangiogenic agents may improve the cancer-eliminating effect of immunotherapy. Because vascular epithelial growth factor has been known to prevent dendritic cell maturation and to modulate inhibitor checkpoints on CD8+ T cells in tumors, targeted antiangiogenic agents may also have immunostimulatory effects.^37^^,^^38^ In addition, it has been hypothesized that antiangiogenic pharmaceutical agents may act synergistically with immunotherapy.^39^ Perhaps, by improving the tumor microenvironment of patients receiving adjuvant atezolizumab, bevacizumab may be associated with better intracranial and extracranial progression-free survivals, especially in those patients with tumors having a PD-L1 of 1% to 49% or those found to be at high risk for brain metastases.

Prospective studies could also be conducted in patients with metastatic disease. Before the current immunotherapy era, four prospective randomized trials have compared stereotactic radiosurgery to WBRT and stereotactic radiosurgery in patients with 1 to 3^40^, ^41^, ^42^ or 1 to 4 brain metastases.^43^ Most patients in these studies had NSCLC (52%–72.1%). All studies did not reveal a survival benefit to WBRT. Despite better intracranial control, all studies revealed that radiosurgical treatment alone was best because of the concerns of neurologic toxicity and the lack of any survival benefit associated with WBRT. Nevertheless, the results of radiosurgical treatment alone needs further improvement owing to local failure rates of approximately 30% (27.2%–33%) and high rates of distal failure (30.1%–63.7%) that was noted in these prospective trials. Perhaps, regimens such as the IMpower150 ABCP arm can provide better intracranial progression-free survival in addition to its extracranial progression-free survival in patients who present with brain metastases.^44^

## Conclusions

Our investigation reveals that patients treated with surgery and postoperative systemic therapy have a risk of brain metastases that approaches 10%. The addition of bevacizumab to adjuvant chemotherapy reduces the risk of the development of all brain metastases and isolated brain metastases as a first recurrence, but it was associated with a numerically shorter survival compared with the control arm. Although many past reviews have revealed that NS histologies are associated with brain metastases from NSCLC, we have reported that the receipt of adjuvant chemotherapy with bevacizumab was associated with a reduction in brain metastases and is a new finding for early stage lung cancer. Because past studies of prophylactic cranial radiation have decreased the incidence of brain metastases while not significantly increasing survival, the assessment of bevacizumab in the adjuvant setting combined with other modalities such as chemotherapy and even immunotherapy in light of recent positive adjuvant data may be worthy of further exploration to reveal whether it can decrease the incidence of brain metastases.

## CRediT Authorship Contribution Statement

**John M. Varlotto, Zhuoxin Sun, Suresh Ramalingam, Heather A. Wakelee:** Conceptualization.

**Zhuoxin Sun, Yating Wang:** Data curation.

**Heather A. Wakelee, John M. Varlotto, Joan Schiller, Zhuoxin Sun, Yating Wang, Suresh Ramalingam:** Formal analysis, Investigation, Methodology, Writing - original draft, Writing – reviewing & editing.

**Heather A. Wakelee:** Funding acquisition.
