# Supplementary material for: Bevacizumab’s Association With a Decreased Risk of Brain Metastases in ECOG-ACRIN E1505, a Phase 3 Randomized Trial of Adjuvant Chemotherapy With or Without Bevacizumab in Surgically Resected NSCLC
Source: JTO Clin Res Rep. 2022 Jan 10;3(3):100274. doi: 10.1016/j.jtocrr.2021.100274 (PMC8908250; doi:10.1016/j.jtocrr.2021.100274)

Supplemental appendix

Table 1

Combined Table 1- Table of demographic factors for total population as well as the 3 recurrent populations. were used to compare the 3 recurrent populations to the population without that type of recurrence

| Variable |  | Total Patients | All Brain Recurrence(ABR) | p-value ^1^ | Extracranial Recurrence  (ECR) | p-value^2^ | Isolated Brain Recurrence (IBR) | p-value ^3^ |
| --- | --- | --- | --- | --- | --- | --- | --- | --- |
| Patient# |  | 1501 | 122 | -- | 472 | -- | 84 | -- |
| Age | Mean (SD) | 60.8(8.8) | 59.8(8.4) | 0.168 | 60.9(8.7) | 0.801 | 60.1(8.0) | 0.513 |
|  | Median (Q1,Q3) | 61 (55,67) | 60 (53,66) |  | 61 (55,67) |  | 62 (56,66) |  |
|  | [Min, Max] | [30,86] | [40,79] |  | [37,84] |  | [40,79] |  |
|  | Freq. of Missing | 0 | 0 |  | 0 |  | 0 |  |
| Sex | Male | 746(50) | 54(44) | 0.22 | 227(48) | 0.405 | 40(48) | 0.737 |
|  | Female | 755(50) | 68(56) |  | 245(52) |  | 44(52) |  |
|  | Unknown/Missing | 0 | 0 |  | 0 |  | 0 |  |
| Race | White | 1302(88) | 110(90) | 0.814 | 403(86) | 0.374 | 77(92) | 0.538 |
|  | Black | 131(9) | 10(8) |  | 50(11) |  | 7(8) |  |
|  | Asian | 38(3) | 2(2) |  | 11(2) |  | 0(0) |  |
|  | Native Hawaiian | 5(0) | 0(0) |  | 1(0) |  | 0(0) |  |
|  | Native American | 6(0) | 0(0) |  | 3(1) |  | 0(0) |  |
|  | Unknown/Missing | 19 | 0 |  | 4 |  | 0 |  |
| Ethnicity | Hispanic | 48(3) | 1(1) | 0.173 | 12(3) | 0.347 | 1(1) | 0.516 |
|  | Non-Hispanic | 1368(97) | 113(99) |  | 437(97) |  | 78(99) |  |
|  | Unknown/Missing | 85 | 8 |  | 23 |  | 5 |  |
| Chemotherapy | Cis/Vinorelbine | 377(25) | 37(30) | 0.543 | 111(24) | 0.562 | 22(26) | 0.404 |
|  | Cis/Docetaxel | 343(23) | 28(23) |  | 103(22) |  | 23(27) |  |
|  | Cis/Gemcitabine | 283(19) | 21(17) |  | 92(19) |  | 18(21) |  |
|  | Cis/Pemetrexed | 497(33) | 36(30) |  | 166(35) |  | 21(25) |  |
|  | Unknown/Missing | 1 | 0 |  | 0 |  | 0 |  |
| Histology | Squamous | 422(28) | 22(18) | **0.03** | 95(20) | **<0.01** | 17(20) | **0.148** |
|  | Adenocarcinoma | 874(58) | 80(66) |  | 309(65) |  | 54(64) |  |
|  | Large cell | 38(3) | 6(5) |  | 10(2) |  | 4(5) |  |
|  | BAC | 13(1) | 0(0) |  | 5(1) |  | 0(0) |  |
|  | NOS | 40(3) | 6(5) |  | 14(3) |  | 5(6) |  |
|  | Combined/Mixed | 93(6) | 8(7) |  | 29(6) |  | 4(5) |  |
|  | Other | 20(1) | 0(0) |  | 10(2) |  | 0(0) |  |
|  | Unknown/Missing | 1 | 0 |  | 0 |  | 0 |  |
| Stage (by Sx Eval) | IB T2N0 | 383(26) | 28(23) | 0.296 | 83(18) | **<0.01** | 22(27) | 0.66 |
|  | IIA T1N1 | 174(12) | 12(10) |  | 43(9) |  | 7(9) |  |
|  | IIB T2N1 | 394(27) | 40(33) |  | 126(27) |  | 28(34) |  |
|  | IIB T3N0 | 68(5) | 4(3) |  | 19(4) |  | 3(4) |  |
|  | IIIA T1N2 | 115(8) | 9(8) |  | 42(9) |  | 5(6) |  |
|  | IIIA T2N2 | 243(17) | 25(21) |  | 116(25) |  | 15(18) |  |
|  | IIIA T3N2 | 20(1) | 1(1) |  | 7(2) |  | 1(1) |  |
|  | IIIA T3N1 | 61(4) | 1(1) |  | 23(5) |  | 1(1) |  |
|  | Unknown/Missing | 43 | 2 |  | 13 |  | 2 |  |
| Weight loss | <5% | 1186(79) | 99(81) | 0.95 | 380(81) | 0.604 | 67(80) | 0.974 |
|  | 5-<10% | 209(14) | 15(12) |  | 62(13) |  | 11(13) |  |
|  | 10-<20% | 91(6) | 7(6) |  | 27(6) |  | 5(6) |  |
|  | > 20% | 12(1) | 1(1) |  | 2(0) |  | 1(1) |  |
|  | Unknown/Missing | 3 | 0 |  | 1 |  | 0 |  |
| PS | Fully active | 879(59) | 70(57) | 0.774 | 276(58) | 0.955 | 45(54) | 0.362 |
|  | Ambulatory | 620(41) | 52(43) |  | 196(42) |  | 39(46) |  |
|  | Unknown/Missing | 2 | 0 |  | 0 |  | 0 |  |
| Pathologic Node Stages | PN0 | 441(32) | 28(27) | 0.411 | 93(22) | **< 0.01** | 22(31) | 0.91 |
|  | PN1 | 589(43) | 46(44) |  | 176(42) |  | 32(45) |  |
|  | PN2 | 351(25) | 31(30) |  | 150(36) |  | 17(24) |  |
|  | Unknown/Missing | 120 | 17 |  | 53 |  | 13 |  |
| Tumor Location | Upper lobe, R | 462(31) | 35(29) | 0.293 | 134(28) | 0.666 | 26(31) | 0.446 |
|  | Middle lobe, R | 69(5) | 8(7) |  | 26(6) |  | 7(8) |  |
|  | Lower lobe, R | 267(18) | 25(20) |  | 85(18) |  | 19(23) |  |
|  | Upper lobe, L | 420(28) | 34(28) |  | 129(27) |  | 21(25) |  |
|  | Lingula | 3(0) | 0(0) |  | 1(0) |  | 0(0) |  |
|  | Lower lobe, L | 222(15) | 17(14) |  | 77(16) |  | 9(11) |  |
|  | Other | 57(4) | 3(2) |  | 20(4) |  | 2(2) |  |
|  | Unknown/Missing | 1 | 0 |  | 0 |  | 0 |  |
| Tumor Size | Mean (SD) | 4.67(2.66) | 4.82(2.45) | 0.293 | 4.85(3.03) | 0.953 | 4.97(2.29) | **0.078** |
|  | Median (Q1,Q3) | 4.2 (2.8,6.0) | 4.5 (3.0,6.5) |  | 4.1 (2.7,6.0) |  | 4.7 (3.2,6.1) |  |
|  | [Min, Max] | [0.4,28.0] | [0.8,13.0] |  | [0.8,24.0] |  | [0.8,11.0] |  |
|  | Freq. of Missing | 2 | 0 |  | 0 |  | 0 |  |
| Resection Type | Intraperi. pneumonectomy | 8(1) | 1(1) | 0.245 | 4(1) | 0.382 | 1(1) | 0.586 |
|  | Pneumonectomy | 184(12) | 22(18) |  | 58(12) |  | 15(18) |  |
|  | Lobectomy | 1134(76) | 88(72) |  | 355(75) |  | 60(71) |  |
|  | Bilobectomy | 105(7) | 5(4) |  | 33(7) |  | 4(5) |  |
|  | Sleeve lobectomy | 22(1) | 3(2) |  | 3(1) |  | 2(2) |  |
|  | Lobectomy and chest wall resect. | 31(2) | 1(1) |  | 13(3) |  | 1(1) |  |
|  | Other | 16(1) | 2(2) |  | 6(1) |  | 1(1) |  |
|  | Unknown/Missing | 1 | 0 |  | 0 |  | 0 |  |
| LN Dissection Type | None | 1(0) | 0(0) | 0.325 | 0(0) | 0.316 | 0(0) | 0.35 |
|  | Incomplete sampling | 89(6) | 12(10) |  | 20(4) |  | 7(8) |  |
|  | Systematic sampling | 689(46) | 58(48) |  | 227(48) |  | 44(52) |  |
|  | Complete MLND | 701(47) | 50(41) |  | 220(47) |  | 31(37) |  |
|  | Other | 19(1) | 2(2) |  | 5(1) |  | 2(2) |  |
|  | Unknown/Missing | 2 | 0 |  | 0 |  | 0 |  |
| Hx Cardiovasc. Disease | No | 1331(89) | 109(89) | 1 | 418(89) | 0.93 | 73(87) | 0.593 |
|  | Yes | 169(11) | 13(11) |  | 54(11) |  | 11(13) |  |
|  | Unknown/Missing | 1 | 0 |  | 0 |  | 0 |  |
| Hx HTN | No | 718(48) | 61(50) | 0.637 | 237(50) | 0.221 | 39(46) | 0.823 |
|  | Yes | 782(52) | 61(50) |  | 235(50) |  | 45(54) |  |
|  | Unknown/Missing | 1 | 0 |  | 0 |  | 0 |  |
| Hx Thrombotic Events | No | 1482(99) | 120(98) | 0.653 | 468(99) | 0.457 | 83(99) | 1 |
|  | Yes | 18(1) | 2(2) |  | 4(1) |  | 1(1) |  |
|  | Unknown/Missing | 1 | 0 |  | 0 |  | 0 |  |
| Hx Wound Compl. | No | 1491(99) | 122(100) | 1 | 468(99) | 0.475 | 84(100) | 1 |
|  | Yes | 9(1) | 0(0) |  | 4(1) |  | 0(0) |  |
|  | Unknown/Missing | 1 | 0 |  | 0 |  | 0 |  |
| CVD-Myocardial Infarction | No | 165(76) | 13(72) | 0.772 | 55(79) | 0.732 | 11(73) | 0.757 |
|  | Yes | 51(24) | 5(28) |  | 15(21) |  | 4(27) |  |
|  | Unknown/Missing | 1285 | 104 |  | 402 |  | 69 |  |
| CVD-Stable Angina | No | 197(92) | 15(88) | 0.631 | 63(90) | 0.432 | 12(86) | 0.307 |
|  | Yes | 17(8) | 2(12) |  | 7(10) |  | 2(14) |  |
|  | Unknown/Missing | 1287 | 105 |  | 402 |  | 70 |  |
| CVD-Unstable Angina | No | 208(97) | 16(94) | 0.395 | 67(96) | 0.395 | 13(93) | 0.337 |
|  | Yes | 6(3) | 1(6) |  | 3(4) |  | 1(7) |  |
|  | Unknown/Missing | 1287 | 105 |  | 402 |  | 70 |  |
| CVD-Other | No | 89(42) | 6(38) | 0.795 | 30(44) | 0.766 | 6(46) | 0.78 |
|  | Yes | 121(58) | 10(62) |  | 38(56) |  | 7(54) |  |
|  | Unknown/Missing | 1291 | 106 |  | 404 |  | 71 |  |
| High baseline BP | No | 1468(98) | 122(100) | 0.264 | 461(98) | 0.832 | 84(100) | 0.396 |
|  | Yes | 26(2) | 0(0) |  | 9(2) |  | 0(0) |  |
|  | Unknown/Missing | 7 | 0 |  | 2 |  | 0 |  |
| Any Anti-Hypertensive Drugs | No | 719(48) | 61(50) | 0.638 | 235(50) | 0.344 | 41(49) | 0.911 |
|  | Yes | 782(52) | 61(50) |  | 237(50) |  | 43(51) |  |
| Smoke after Dx | No | 694(60) | 49(53) | 0.333 | 228(62) | 0.372 | 34(51) | 0.256 |
|  | Yes | 467(40) | 43(47) |  | 136(37) |  | 33(49) |  |
|  | Refused to answer | 5(0) | 0(0) |  | 2(1) |  | 0(0) |  |
|  | Unknown/Missing | 335 | 30 |  | 106 |  | 17 |  |
| Currently smoking | No | 1176(88) | 94(85) | 0.361 | 368(90) | 0.17 | 67(85) | 0.371 |
|  | Yes | 160(12) | 16(15) |  | 41(10) |  | 12(15) |  |
|  | Unknown/Missing | 165 | 12 |  | 63 |  | 5 |  |
| Cigarettes per day | Mean (SD) | 24.0(12.8) | 24.2(10.8) | 0.581 | 23.3(13.0) | **0.062** | 25.2(10.8) | 0.167 |
|  | Median (Q1,Q3) | 20 (20,30) | 20 (20,30) |  | 20 (15,30) |  | 20 (20,30) |  |
|  | [Min, Max] | [0,100] | [1,60] |  | [0,80] |  | [1,60] |  |
|  | Freq. of Missing | 181 | 15 |  | 67 |  | 5 |  |
| Bevacizumab | Without Bevacizumab | 749(50) | 74(61) | **0.014** | 232(49) | 0.698 | 52(62) | **0.025** |
|  | With Bevacizumab | 752(50) | 48(39) |  | 240(51) |  | 32(38) |  |
|  | Unknown/Missing | 0 | 0 |  | 0 |  | 0 |  |
| Percentage of N1 positive | Mean (SD) | 0.1(0.2) | 0.2(0.3) | 0.363 | 0.2(0.3) | **<0.01** | 0.1(0.3) | 0.64 |
|  | Median (Q1,Q3) | 0 (0,0) | 0 (0,0) |  | 0 (0,0) |  | 0 (0,0) |  |
|  | [Min, Max] | [0,1] | [0,1] |  | [0,1] |  | [0,1] |  |
|  | Freq. of Missing | 53 | 7 |  | 18 |  | 4 |  |
| Percentage of N2 positive | Mean (SD) | 0.4(0.4) | 0.5(0.4) | 0.192 | 0.5(0.4) | **<0.01** | 0.4(0.4) | 0.614 |
|  | Median (Q1,Q3) | 0 (0,1) | 0 (0,1) |  | 0 (0,1) |  | 0 (0,1) |  |
|  | [Min, Max] | [0,1] | [0,1] |  | [0,1] |  | [0,1] |  |
|  | Freq. of Missing | 145 | 12 |  | 42 |  | 8 |  |

1: P-values to compare patients with brain mets vs. without brain mets based on univariate analysis.

2: P-values to compare patients with extracranial recurrence vs. without extracranial recurrence based on univariate analysis

3: P-values to compare patients with isolated brain mets vs. without isolated brain mets based on univariate analysis.

Table 2 Risk Factors associated with All Brain Recurrence (ABR)

| Variable | Category | No CNS Recurrence | CNS Recurrence | All patients | p-value |
| --- | --- | --- | --- | --- | --- |
| Patient# |  | 1379 | 122 | 1501 | - |
| Age | Mean (SD)  Median (Q1,Q3)  [Min, Max]  Freq. of Missing | 60.8(8.9)  61 (55,67)  [30,86]  0 | 59.8(8.4)  60 (53,66)  [40,79]  0 | 60.8(8.8)  61 (55,67)  [30,86]  0 | 0.168 |
| Sex | Male  Female  Unknown | 692(50)  687(50)  0 | 54(44)  68(56) | 746(50)  755(50) | 0.22 |
| Race | White | 1192(88) | 110(90) | 1302(88) | 0.814 |
|  | Black | 121(9) | 10(8) | 131(9) |  |
|  | Asian | 36(3) | 2(2) | 38(3) |  |
|  | Native Hawaiian | 5(0) | 0(0) | 5(0) |  |
|  | Native American | 6(0) | 0(0) | 6(0) |  |
|  | Unknown/Missing | 19 | 0 | 19 |  |
| Ethnicity | Hispanic | 47(4) | 1(1) | 48(3) | 0.173 |
|  | Non-Hispanic | 1255(96) | 113(99) | 1368(97) |  |
|  | Unknown/Missing | 77 | 8 | 85 |  |
| Chemotherapy | Cis/Vinorelbine  Cis/Docetaxel  Cis/Gemcitabine  Cis/Pemetrexed  Unknown | 340(25)  315(23)  262(19)  461(34)  1 | 37(31)  28(23)  21(17)  36(29)  0 | 377(25) –  343(23)  283(19)  497(33)  1 | 0.543 |
| **Histology** | Squamous  Adenocarcinoma  Large cell  BAC  NOS  Combined/Mixed  Other  Unknown | 400(29)  794(58)  32(2)  13(1)  34(2)  85(6)  20(1)  1 | 22(18)  80(65)  6(5)  0(0)  6(5)  8(7)  0(0)  0 | 422(28)  874(58)  38(3)  13(1)  40(3)  93(6)  20(1)  1 | 0.025 |
| Stage | IB T2N0  IIA T1N1  IIB T2N1  IIB T3N0  IIIA T1N2  IIIA T2N2  IIIA T3N2  IIIA T3N1  Unknown | 355(27)  162(12)  354(26)  64(5)  106(8)  218(16)  19(1)  60(5)  41 | 28(23)  12(10)  40(34)  4(3)  9(8)  25(21)  1(1)  1(1)  2 | 383(26)  174(12)  394(27)  68(5)  115(8)  243(17)  20(1)  61(4)  43 | 0.296 |
| Weight loss | <5%  5-<10%  10-<20%  > 20%  Unknown | 1087(79)  194(14)  84(6)  11(1)  3 | 99(81)  15(12)  7(6)  1(1)  0 | 1186(79)  209(14)  91(6)  12(1)  3 | 0.95 |
| PS | Fully active | 809(59) | 70(57) | 879(59) | 0.774 |
|  | Ambulatory | 568(41) | 52(43) | 620(41) |  |
|  | Unknown/Missing | 2 | 0 | 2 |  |
| Pathologic Node Stages | PN0 | 413(32) | 28(27) | 441(32) | 0.411 |
|  | PN1 | 543(43) | 46(44) | 589(43) |  |
|  | PN2 | 320(25) | 31(30) | 351(25) |  |
|  | Unknown/Missing | 103 | 17 | 120 |  |
| Tumor Location | Upper lobe, R | 427(31) | 35(29) | 462(31) | 0.833 |
|  | Middle lobe, R | 61(4) | 8(7) | 69(5) |  |
|  | Lower lobe, R | 242(18) | 25(20) | 267(18) |  |
|  | Upper lobe, L | 386(28) | 34(28) | 420(28) |  |
|  | Lingula | 3(0) | 0(0) | 3(0) |  |
|  | Lower lobe, L | 205(15) | 17(14) | 222(15) |  |
|  | Other | 54(4) | 3(2) | 57(4) |  |
|  | Unknown/Missing | 1 | 0 | 1 |  |
| Tumor Size | Mean (SD)  Median (Q1,Q3)  [Min, Max]  Freq. of Missing | 4.66(2.68)  4.2 (2.8,6.0)  [0.4,28.0]  2 | 4.82(2.45)  4.5(3.0,6.5)  [0.8,13.0]  0 | 4.67(2.66)  4.2 (2.8,6.0)  [0.4,28.0]  2 | 0.293 |
| Resection Type | Intraperi. Pneumo  Pneumonectomy  Lobectomy  Bilobectomy  Sleeve lobectomy  Lobect/chestwall  Other  Unknown | 7(1)  162(12)  1046(76)  100(7)  19(1)  30(2)  14(1)  1 | 1(1)  22(17)  88(72)  5(4)  3(2)  1(1)  2(2)  0 | 8(1)  184(12)  1134(76)  105(7)  22(1)  31(2)  16(1)  1 | 0.245 |
| LN Dissection Type | None  Incompl sampl  System sampling  Complete MLND  Other  Unknown | 1(0)  77(6)  631(46)  651(47)  17(1)  2 | 0(0)  12(10)  58(48)  50(41)  2(2)  0 | 1(0)  89(6)  689(46)  701(47)  19(1)  2 | 0.325 |
| Hx Cardiovasc. Disease | No | 1222(89) | 109(89) | 1331(89) | 1 |
|  | Yes | 156(11) | 13(11) | 169(11) |  |
|  | Unknown/Missing | 1 | 0 | 1 |  |
| Hx HTN | No | 657(48) | 61(50) | 718(48) | 0.637 |
|  | Yes | 721(52) | 61(50) | 782(52) |  |
|  | Unknown/Missing | 1 | 0 | 1 |  |
| Hx Thrombotic Events | No | 1362(99) | 120(98) | 1482(99) | 0.653 |
|  | Yes | 16(1) | 2(2) | 18(1) |  |
|  | Unknown/Missing | 1 | 0 | 1 |  |
| Hx Wound Compl. | No | 1369(99) | 122(100) | 1491(99) | 1 |
|  | Yes | 9(1) | 0(0) | 9(1) |  |
|  | Unknown/Missing | 1 | 0 | 1 |  |
| CVD-Myocardial Infarction | No | 152(77) | 13(72) | 165(76) | 0.772 |
|  | Yes | 46(23) | 5(28) | 51(24) |  |
|  | Unknown/Missing | 1181 | 104 | 1285 |  |
| CVD-Stable Angina | No | 182(92) | 15(88) | 197(92) | 0.631 |
|  | Yes | 15(8) | 2(12) | 17(8) |  |
|  | Unknown/Missing | 1182 | 105 | 1287 |  |
| CVD-Unstable Angina | No | 192(97) | 16(94) | 208(97) | 0.395 |
|  | Yes | 5(3) | 1(6) | 6(3) |  |
|  | Unknown/Missing | 1182 | 105 | 1287 |  |
| CVD-Other | No | 83(43) | 6(38) | 89(42) | 0.795 |
|  | Yes | 111(57) | 10(62) | 121(58) |  |
|  | Unknown/Missing | 1185 | 106 | 1291 |  |
| High baseline BP | No | 1346(98) | 122(100) | 1468(98) | 0.264 |
|  | Yes | 26(2) | 0(0) | 26(2) |  |
|  | Unknown/Missing | 7 | 0 | 7 |  |
| Smoke after Dx | No | 645(60) | 49(53) | 694(60) | 0.333 |
|  | Yes | 424(39) | 43(47) | 467(40) |  |
|  | Refused to answer | 5(0) | 0(0) | 5(0) |  |
|  | Unknown/Missing | 305 | 30 | 335 |  |
| Currently smoking | No | 1082(88) | 94(85) | 1176(88) | 0.361 |
|  | Yes | 144(12) | 16(15) | 160(12) |  |
|  | Unknown/Missing | 153 | 12 | 165 |  |
| Cigarettes per day | Mean (SD) | 24.0(12.9) | 24.2(10.8) | 24.0(12.8) | 0.581 |
|  | Median (Q1,Q3) | 20 (20,30) | 20 (20,30) | 20 (20,30) |  |
|  | [Min, Max] | [0,100] | [1,60] | [0,100] |  |
|  | Freq. of Missing | 166 | 15 | 181 |  |
| **Bevacizumab** | Without Bevacizumab | 675(49) | 74(61) | 749(50) | 0.014 |
|  | With Bevacizumab | 704(51) | 48(39) | 752(50) |  |
|  | Unknown/Missing | 0 | 0 | 0 |  |
| Percentage of N1 positive | Mean (SD) | 0.1(0.2) | 0.2(0.3) | 0.1(0.2) | 0.363 |
|  | Median (Q1,Q3) | 0 (0,0) | 0 (0,0) | 0 (0,0) |  |
|  | [Min, Max] | [0,1] | [0,1] | [0,1] |  |
|  | Freq. of Missing | 46 | 7 | 53 |  |
| Percentage of N2 positive | Mean (SD) | 0.4(0.4) | 0.5(0.4) | 0.4(0.4) | 0.192 |
|  | Median (Q1,Q3) | 0 (0,1) | 0 (0,1) | 0 (0,1) |  |
|  | [Min, Max] | [0,1] | [0,1] | [0,1] |  |
|  | Freq. of Missing | 133 | 12 | 145 |  |
| Any Anti-Hypertensive Drugs | No | 658(48) | 61(50) | 719(48) | 0.638 |
|  | Yes | 721(52) | 61(50) | 782(52) |  |

Supplemental Table 3 UNIVARIATE ANALYSIS for Isolated Brain Recurrence #(%)

| Variable |  | No Isolated Brain Recurrence | Isolated Brain Recurrence | Total | p-value |
| --- | --- | --- | --- | --- | --- |
| Patient # |  | 1417 | 84 | 1501 | -- |
| Age | Mean (SD) | 60.8(8.9) | 60.1(8.0) | 60.8(8.8) | 0.513 |
|  | Median (Q1,Q3) | 61 (55,67) | 62 (56,66) | 61 (55,67) |  |
|  | [Min, Max] | [30,86] | [40,79] | [30,86] |  |
|  | Freq. of Missing | 0 | 0 | 0 |  |
| Sex | Male | 706(50) | 40(48) | 746(50) | 0.737 |
|  | Female | 711(50) | 44(52) | 755(50) |  |
|  | Unknown/Missing | 0 | 0 | 0 |  |
| Race | White | 1225(88) | 77(92) | 1302(88) | 0.538 |
|  | Black | 124(9) | 7(8) | 131(9) |  |
|  | Asian | 38(3) | 0(0) | 38(3) |  |
|  | Native Hawaiian | 5(0) | 0(0) | 5(0) |  |
|  | Native American | 6(0) | 0(0) | 6(0) |  |
|  | Unknown/Missing | 19 | 0 | 19 |  |
| Ethnicity | Hispanic | 47(4) | 1(1) | 48(3) | 0.516 |
|  | Non-Hispanic | 1290(96) | 78(99) | 1368(97) |  |
|  | Unknown/Missing | 80 | 5 | 85 |  |
| Chemotherapy | Cis/Vinorelbine | 355(25) | 22(26) | 377(25) | 0.404 |
|  | Cis/Docetaxel | 320(23) | 23(27) | 343(23) |  |
|  | Cis/Gemcitabine | 265(19) | 18(21) | 283(19) |  |
|  | Cis/Pemetrexed | 476(34) | 21(25) | 497(33) |  |
|  | Unknown/Missing | 1 | 0 | 1 |  |
| Histology | Squamous | 405(29) | 17(20) | 422(28) | 0.148 |
|  | Adenocarcinoma | 820(58) | 54(64) | 874(58) |  |
|  | Large cell | 34(2) | 4(5) | 38(3) |  |
|  | BAC | 13(1) | 0(0) | 13(1) |  |
|  | NOS | 35(2) | 5(6) | 40(3) |  |
|  | Combined/Mixed | 89(6) | 4(5) | 93(6) |  |
|  | Other | 20(1) | 0(0) | 20(1) |  |
|  | Unknown/Missing | 1 | 0 | 1 |  |
| Stage (by Sx Eval) | IB T2N0 | 361(26) | 22(27) | 383(26) | 0.66 |
|  | IIA T1N1 | 167(12) | 7(9) | 174(12) |  |
|  | IIB T2N1 | 366(27) | 28(34) | 394(27) |  |
|  | IIB T3N0 | 65(5) | 3(4) | 68(5) |  |
|  | IIIA T1N2 | 110(8) | 5(6) | 115(8) |  |
|  | IIIA T2N2 | 228(17) | 15(18) | 243(17) |  |
|  | IIIA T3N2 | 19(1) | 1(1) | 20(1) |  |
|  | IIIA T3N1 | 60(4) | 1(1) | 61(4) |  |
|  | Unknown/Missing | 41 | 2 | 43 |  |
| Weight loss | <5% | 1119(79) | 67(80) | 1186(79) | 0.974 |
|  | 5-<10% | 198(14) | 11(13) | 209(14) |  |
|  | 10-<20% | 86(6) | 5(6) | 91(6) |  |
|  | > 20% | 11(1) | 1(1) | 12(1) |  |
|  | Unknown/Missing | 3 | 0 | 3 |  |
| PS | Fully active | 834(59) | 45(54) | 879(59) | 0.362 |
|  | Ambulatory | 581(41) | 39(46) | 620(41) |  |
|  | Unknown/Missing | 2 | 0 | 2 |  |
| Pathologic Node Stages | PN0 | 419(32) | 22(31) | 441(32) | 0.91 |
|  | PN1 | 557(43) | 32(45) | 589(43) |  |
|  | PN2 | 334(25) | 17(24) | 351(25) |  |
|  | Unknown/Missing | 107 | 13 | 120 |  |
| Tumor Location | Upper lobe, R | 436(31) | 26(31) | 462(31) | 0.446 |
|  | Middle lobe, R | 62(4) | 7(8) | 69(5) |  |
|  | Lower lobe, R | 248(18) | 19(23) | 267(18) |  |
|  | Upper lobe, L | 399(28) | 21(25) | 420(28) |  |
|  | Lingula | 3(0) | 0(0) | 3(0) |  |
|  | Lower lobe, L | 213(15) | 9(11) | 222(15) |  |
|  | Other | 55(4) | 2(2) | 57(4) |  |
|  | Unknown/Missing | 1 | 0 | 1 |  |
| Tumor Size | Mean (SD) | 4.66(2.68) | 4.97(2.29) | 4.67(2.66) | 0.078 |
|  | Median (Q1,Q3) | 4.2 (2.8,6.0) | 4.7 (3.2,6.1) | 4.2 (2.8,6.0) |  |
|  | [Min, Max] | [0.4,28.0] | [0.8,11.0] | [0.4,28.0] |  |
|  | Freq. of Missing | 2 | 0 | 2 |  |
| Resection Type | Intraperi. pneumonectomy | 7(0) | 1(1) | 8(1) | 0.586 |
|  | Pneumonectomy | 169(12) | 15(18) | 184(12) |  |
|  | Lobectomy | 1074(76) | 60(71) | 1134(76) |  |
|  | Bilobectomy | 101(7) | 4(5) | 105(7) |  |
|  | Sleeve lobectomy | 20(1) | 2(2) | 22(1) |  |
|  | Lobectomy and chest wall resect. | 30(2) | 1(1) | 31(2) |  |
|  | Other | 15(1) | 1(1) | 16(1) |  |
|  | Unknown/Missing | 1 | 0 | 1 |  |
| LN Dissection Type | None | 1(0) | 0(0) | 1(0) | 0.35 |
|  | Incomplete sampling | 82(6) | 7(8) | 89(6) |  |
|  | Systematic sampling | 645(46) | 44(52) | 689(46) |  |
|  | Complete MLND | 670(47) | 31(37) | 701(47) |  |
|  | Other | 17(1) | 2(2) | 19(1) |  |
|  | Unknown/Missing | 2 | 0 | 2 |  |
| Hx Cardiovasc. Disease | No | 1258(89) | 73(87) | 1331(89) | 0.593 |
|  | Yes | 158(11) | 11(13) | 169(11) |  |
|  | Unknown/Missing | 1 | 0 | 1 |  |
| Hx HTN | No | 679(48) | 39(46) | 718(48) | 0.823 |
|  | Yes | 737(52) | 45(54) | 782(52) |  |
|  | Unknown/Missing | 1 | 0 | 1 |  |
| Hx Thrombotic Events | No | 1399(99) | 83(99) | 1482(99) | 1 |
|  | Yes | 17(1) | 1(1) | 18(1) |  |
|  | Unknown/Missing | 1 | 0 | 1 |  |
| Hx Wound Compl. | No | 1407(99) | 84(100) | 1491(99) | 1 |
|  | Yes | 9(1) | 0(0) | 9(1) |  |
|  | Unknown/Missing | 1 | 0 | 1 |  |
| CVD-Myocardial Infarction | No | 154(77) | 11(73) | 165(76) | 0.757 |
|  | Yes | 47(23) | 4(27) | 51(24) |  |
|  | Unknown/Missing | 1216 | 69 | 1285 |  |
| CVD-Stable Angina | No | 185(92) | 12(86) | 197(92) | 0.307 |
|  | Yes | 15(8) | 2(14) | 17(8) |  |
|  | Unknown/Missing | 1217 | 70 | 1287 |  |
| CVD-Unstable Angina | No | 195(98) | 13(93) | 208(97) | 0.337 |
|  | Yes | 5(2) | 1(7) | 6(3) |  |
|  | Unknown/Missing | 1217 | 70 | 1287 |  |
| CVD-Other | No | 83(42) | 6(46) | 89(42) | 0.78 |
|  | Yes | 114(58) | 7(54) | 121(58) |  |
|  | Unknown/Missing | 1220 | 71 | 1291 |  |
| High baseline BP | No | 1384(98) | 84(100) | 1468(98) | 0.396 |
|  | Yes | 26(2) | 0(0) | 26(2) |  |
|  | Unknown/Missing | 7 | 0 | 7 |  |
| Any Anti-Hypertensive Drugs | No | 678(48) | 41(49) | 719(48) | 0.911 |
|  | Yes | 739(52) | 43(51) | 782(52) |  |
| Smoke after Dx | No | 660(60) | 34(51) | 694(60) | 0.256 |
|  | Yes | 434(39) | 33(49) | 467(40) |  |
|  | Refused to answer | 5(0) | 0(0) | 5(0) |  |
|  | Unknown/Missing | 318 | 17 | 335 |  |
| Currently smoking | No | 1109(88) | 67(85) | 1176(88) | 0.371 |
|  | Yes | 148(12) | 12(15) | 160(12) |  |
|  | Unknown/Missing | 160 | 5 | 165 |  |
| Cigarettes per day | Mean (SD) | 24.0(12.9) | 25.2(10.8) | 24.0(12.8) | 0.167 |
|  | Median (Q1,Q3) | 20 (20,30) | 20 (20,30) | 20 (20,30) |  |
|  | [Min, Max] | [0,100] | [1,60] | [0,100] |  |
|  | Freq. of Missing | 176 | 5 | 181 |  |
| **Bevacizumab** | Without Bevacizumab | 697(49) | 52(62) | 749(50) | **0.025** |
|  | With Bevacizumab | 720(51) | 32(38) | 752(50) |  |
|  | Unknown/Missing | 0 | 0 | 0 |  |
| Percentage of N1 positive | Mean (SD) | 0.1(0.2) | 0.1(0.3) | 0.1(0.2) | 0.64 |
|  | Median (Q1,Q3) | 0 (0,0) | 0 (0,0) | 0 (0,0) |  |
|  | [Min, Max] | [0,1] | [0,1] | [0,1] |  |
|  | Freq. of Missing | 49 | 4 | 53 |  |
| Percentage of N2 positive | Mean (SD) | 0.4(0.4) | 0.4(0.4) | 0.4(0.4) | 0.614 |
|  | Median (Q1,Q3) | 0 (0,1) | 0 (0,1) | 0 (0,1) |  |
|  | [Min, Max] | [0,1] | [0,1] | [0,1] |  |
|  | Freq. of Missing | 137 | 8 | 145 |  |

Supplemental Table 4 UNIVARIATE ANALYSIS for Extracranial Recurrence #(%)

| Variable |  | No Extracranial Recurrence | Extracranial Recurrence | Total | p-value |
| --- | --- | --- | --- | --- | --- |
| Patient# |  | 1029 | 472 | 1501 | -- |
| Age | Mean (SD) | 60.7(8.9) | 60.9(8.7) | 60.8(8.8) | 0.801 |
|  | Median (Q1,Q3) | 61 (55,67) | 61 (55,67) | 61 (55,67) |  |
|  | [Min, Max] | [30,86] | [37,84] | [30,86] |  |
|  | Freq. of Missing | 0 | 0 | 0 |  |
| Sex | Male | 519(50) | 227(48) | 746(50) | 0.405 |
|  | Female | 510(50) | 245(52) | 755(50) |  |
|  | Unknown/Missing | 0 | 0 | 0 |  |
| Race | White | 899(89) | 403(86) | 1302(88) | 0.374 |
|  | Black | 81(8) | 50(11) | 131(9) |  |
|  | Asian | 27(3) | 11(2) | 38(3) |  |
|  | Native Hawaiian | 4(0) | 1(0) | 5(0) |  |
|  | Native American | 3(0) | 3(1) | 6(0) |  |
|  | Unknown/Missing | 15 | 4 | 19 |  |
| Ethnicity | Hispanic | 36(4) | 12(3) | 48(3) | 0.347 |
|  | Non-Hispanic | 931(96) | 437(97) | 1368(97) |  |
|  | Unknown/Missing | 62 | 23 | 85 |  |
| Chemotherapy | Cis/Vinorelbine | 266(26) | 111(24) | 377(25) | 0.562 |
|  | Cis/Docetaxel | 240(23) | 103(22) | 343(23) |  |
|  | Cis/Gemcitabine | 191(19) | 92(19) | 283(19) |  |
|  | Cis/Pemetrexed | 331(32) | 166(35) | 497(33) |  |
|  | Unknown/Missing | 1 | 0 | 1 |  |
| **Histology** | Squamous | 327(32) | 95(20) | 422(28) | **< 0.01** |
|  | Adenocarcinoma | 565(55) | 309(65) | 874(58) |  |
|  | Large cell | 28(3) | 10(2) | 38(3) |  |
|  | BAC | 8(1) | 5(1) | 13(1) |  |
|  | NOS | 26(3) | 14(3) | 40(3) |  |
|  | Combined/Mixed | 64(6) | 29(6) | 93(6) |  |
|  | Other | 10(1) | 10(2) | 20(1) |  |
|  | Unknown/Missing | 1 | 0 | 1 |  |
| **Stage (by Sx Eval)** | IB T2N0 | 300(30) | 83(18) | 383(26) | **< 0.01** |
|  | IIA T1N1 | 131(13) | 43(9) | 174(12) |  |
|  | IIB T2N1 | 268(27) | 126(27) | 394(27) |  |
|  | IIB T3N0 | 49(5) | 19(4) | 68(5) |  |
|  | IIIA T1N2 | 73(7) | 42(9) | 115(8) |  |
|  | IIIA T2N2 | 127(13) | 116(25) | 243(17) |  |
|  | IIIA T3N2 | 13(1) | 7(2) | 20(1) |  |
|  | IIIA T3N1 | 38(4) | 23(5) | 61(4) |  |
|  | Unknown/Missing | 30 | 13 | 43 |  |
| Weight loss | <5% | 806(78) | 380(81) | 1186(79) | 0.604 |
|  | 5-<10% | 147(14) | 62(13) | 209(14) |  |
|  | 10-<20% | 64(6) | 27(6) | 91(6) |  |
|  | > 20% | 10(1) | 2(0) | 12(1) |  |
|  | Unknown/Missing | 2 | 1 | 3 |  |
| PS | Fully active | 603(59) | 276(58) | 879(59) | 0.955 |
|  | Ambulatory | 424(41) | 196(42) | 620(41) |  |
|  | Unknown/Missing | 2 | 0 | 2 |  |
| **Pathologic Node Stages** | PN0 | 348(36) | 93(22) | 441(32) | **< 0.01** |
|  | PN1 | 413(43) | 176(42) | 589(43) |  |
|  | PN2 | 201(21) | 150(36) | 351(25) |  |
|  | Unknown/Missing | 67 | 53 | 120 |  |
| Tumor Location | Upper lobe, R | 328(32) | 134(28) | 462(31) | 0.666 |
|  | Middle lobe, R | 43(4) | 26(6) | 69(5) |  |
|  | Lower lobe, R | 182(18) | 85(18) | 267(18) |  |
|  | Upper lobe, L | 291(28) | 129(27) | 420(28) |  |
|  | Lingula | 2(0) | 1(0) | 3(0) |  |
|  | Lower lobe, L | 145(14) | 77(16) | 222(15) |  |
|  | Other | 37(4) | 20(4) | 57(4) |  |
|  | Unknown/Missing | 1 | 0 | 1 |  |
| Tumor Size | Mean (SD) | 4.59(2.47) | 4.85(3.03) | 4.67(2.66) | 0.953 |
|  | Median (Q1,Q3) | 4.4 (2.8,6.0) | 4.1 (2.7,6.0) | 4.2 (2.8,6.0) |  |
|  | [Min, Max] | [0.4,28.0] | [0.8,24.0] | [0.4,28.0] |  |
|  | Freq. of Missing | 2 | 0 | 2 |  |
| Resection Type | Intraperi. pneumonectomy | 4(0) | 4(1) | 8(1) | 0.382 |
|  | Pneumonectomy | 126(12) | 58(12) | 184(12) |  |
|  | Lobectomy | 779(76) | 355(75) | 1134(76) |  |
|  | Bilobectomy | 72(7) | 33(7) | 105(7) |  |
|  | Sleeve lobectomy | 19(2) | 3(1) | 22(1) |  |
|  | Lobectomy and chest wall resect. | 18(2) | 13(3) | 31(2) |  |
|  | Other | 10(1) | 6(1) | 16(1) |  |
|  | Unknown/Missing | 1 | 0 | 1 |  |
| LN Dissection Type | None | 1(0) | 0(0) | 1(0) | 0.316 |
|  | Incomplete sampling | 69(7) | 20(4) | 89(6) |  |
|  | Systematic sampling | 462(45) | 227(48) | 689(46) |  |
|  | Complete MLND | 481(47) | 220(47) | 701(47) |  |
|  | Other | 14(1) | 5(1) | 19(1) |  |
|  | Unknown/Missing | 2 | 0 | 2 |  |
| Hx Cardiovasc. Disease | No | 913(89) | 418(89) | 1331(89) | 0.93 |
|  | Yes | 115(11) | 54(11) | 169(11) |  |
|  | Unknown/Missing | 1 | 0 | 1 |  |
| Hx HTN | No | 481(47) | 237(50) | 718(48) | 0.221 |
|  | Yes | 547(53) | 235(50) | 782(52) |  |
|  | Unknown/Missing | 1 | 0 | 1 |  |
| Hx Thrombotic Events | No | 1014(99) | 468(99) | 1482(99) | 0.457 |
|  | Yes | 14(1) | 4(1) | 18(1) |  |
|  | Unknown/Missing | 1 | 0 | 1 |  |
| Hx Wound Compl. | No | 1023(100) | 468(99) | 1491(99) | 0.475 |
|  | Yes | 5(0) | 4(1) | 9(1) |  |
|  | Unknown/Missing | 1 | 0 | 1 |  |
| CVD-Myocardial Infarction | No | 110(75) | 55(79) | 165(76) | 0.732 |
|  | Yes | 36(25) | 15(21) | 51(24) |  |
|  | Unknown/Missing | 883 | 402 | 1285 |  |
| CVD-Stable Angina | No | 134(93) | 63(90) | 197(92) | 0.432 |
|  | Yes | 10(7) | 7(10) | 17(8) |  |
|  | Unknown/Missing | 885 | 402 | 1287 |  |
| CVD-Unstable Angina | No | 141(98) | 67(96) | 208(97) | 0.395 |
|  | Yes | 3(2) | 3(4) | 6(3) |  |
|  | Unknown/Missing | 885 | 402 | 1287 |  |
| CVD-Other | No | 59(42) | 30(44) | 89(42) | 0.766 |
|  | Yes | 83(58) | 38(56) | 121(58) |  |
|  | Unknown/Missing | 887 | 404 | 1291 |  |
| High baseline BP | No | 1007(98) | 461(98) | 1468(98) | 0.832 |
|  | Yes | 17(2) | 9(2) | 26(2) |  |
|  | Unknown/Missing | 5 | 2 | 7 |  |
| Any Anti-Hypertensive Drugs | No | 484(47) | 235(50) | 719(48) | 0.344 |
|  | Yes | 545(53) | 237(50) | 782(52) |  |
| Smoke after Dx | No | 466(58) | 228(62) | 694(60) | 0.372 |
|  | Yes | 331(41) | 136(37) | 467(40) |  |
|  | Refused to answer | 3(0) | 2(1) | 5(0) |  |
|  | Unknown/Missing | 229 | 106 | 335 |  |
| Currently smoking | No | 808(87) | 368(90) | 1176(88) | 0.17 |
|  | Yes | 119(13) | 41(10) | 160(12) |  |
|  | Unknown/Missing | 102 | 63 | 165 |  |
| **Cigarettes per day** | Mean (SD) | 24.3(12.6) | 23.3(13.0) | 24.0(12.8) | **0.062** |
|  | Median (Q1,Q3) | 20 (20,30) | 20 (15,30) | 20 (20,30) |  |
|  | [Min, Max] | [0,100] | [0,80] | [0,100] |  |
|  | Freq. of Missing | 114 | 67 | 181 |  |
| Bevacizumab | Without Bevacizumab | 517(50) | 232(49) | 749(50) | 0.698 |
|  | With Bevacizumab | 512(50) | 240(51) | 752(50) |  |
|  | Unknown/Missing | 0 | 0 | 0 |  |
| **Percentage of N1 positive** | Mean (SD) | 0.1(0.2) | 0.2(0.3) | 0.1(0.2) | **< 0.01** |
|  | Median (Q1,Q3) | 0 (0,0) | 0 (0,0) | 0 (0,0) |  |
|  | [Min, Max] | [0,1] | [0,1] | [0,1] |  |
|  | Freq. of Missing | 35 | 18 | 53 |  |
| **Percentage of N2 positive** | Mean (SD) | 0.4(0.4) | 0.5(0.4) | 0.4(0.4) | **< 0.01** |
|  | Median (Q1,Q3) | 0 (0,1) | 0 (0,1) | 0 (0,1) |  |
|  | [Min, Max] | [0,1] | [0,1] | [0,1] |  |
|  | Freq. of Missing | 103 | 42 | 145 |  |

Supplementary Table 5. All Brain Recurrences(ABR) at 1, 3, and 6 years by histology and administration of bevacizumab.

| Category | Numbers* | Rate of TBR 1 yr | Rate of TBR- 3 yr | Rate of TBR 6 yr |
| --- | --- | --- | --- | --- |
| Non-squamous non-small cell lung cancer without Bevacizumab | 533 | 5.71% | 12.39% | 13.80% |
| Non-squamous non-small cell carcinoma with Bevacizumab | 545 | 2.16% | 7.09% | 9.78% |
| Squamous cell carcinoma without Bevacizumab | 216 | 4.79% | 6.37% | 6.37% |
| Squamous cell carcinoma with Bevacizumab | 206 | 2.14% | 4.88% | 4.88% |

*The total number is 1500, one is missing for NS-NSCLC histology

Table 6A studies investigating the incidence and risk factors associated with brain metastases in patients undergoing definitive local therapy. References: Duke(1), Peking(13), Peking Union(14)


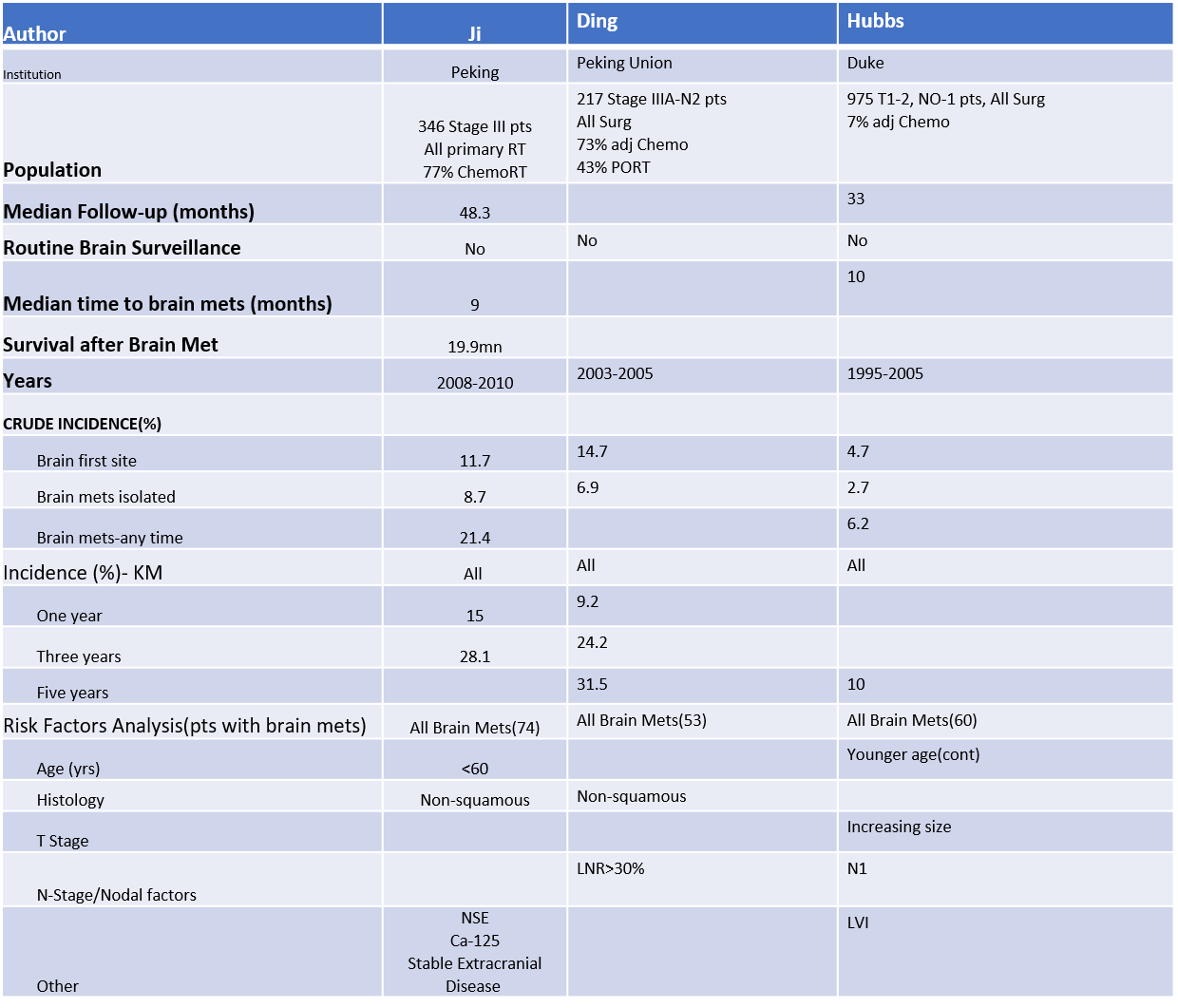


Table 6B studies investigating the incidence and risk factors associated with brain metastastases in patients undergoing definitive local therapy. References: Multi-institutional(2), Harvard(15), Besancon(16), Penn(17), Sun Yat-sen University(18), Goustave Roussy(19), San Rafael(20)


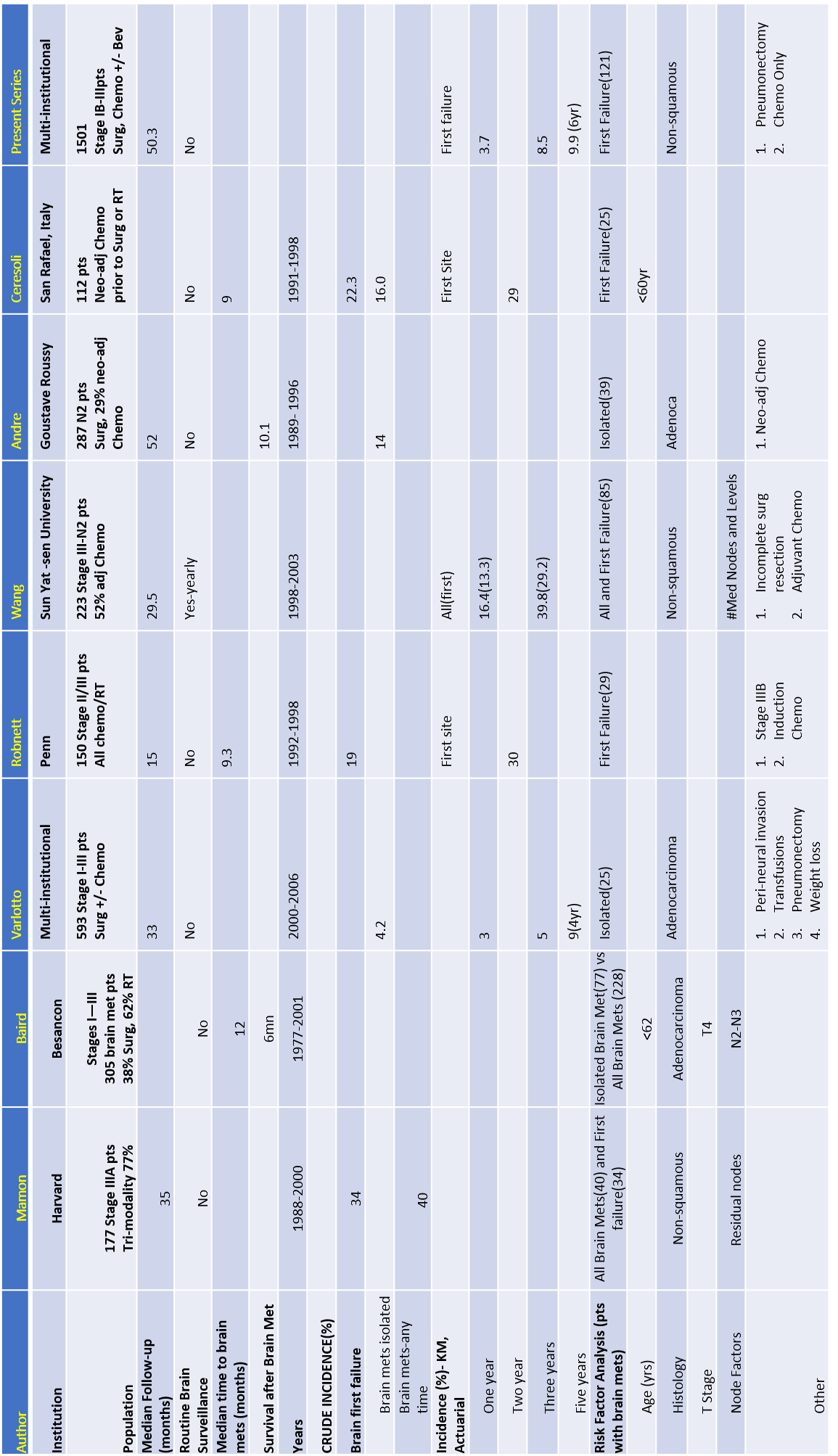


Table 8 studies investigating the incidence and risk factors associated with brain metastases in patients undergoing definitive local therapy from prospective randomized trials of whole brain radiation. Only data for the control arms (no radiation) are listed. References: RTOG-Cox(21), MD Anderson(24), RTOG- Russell(22), RTOG-Sun(23), Guangzhou(25), NVLAT-11/DLCRG(26)


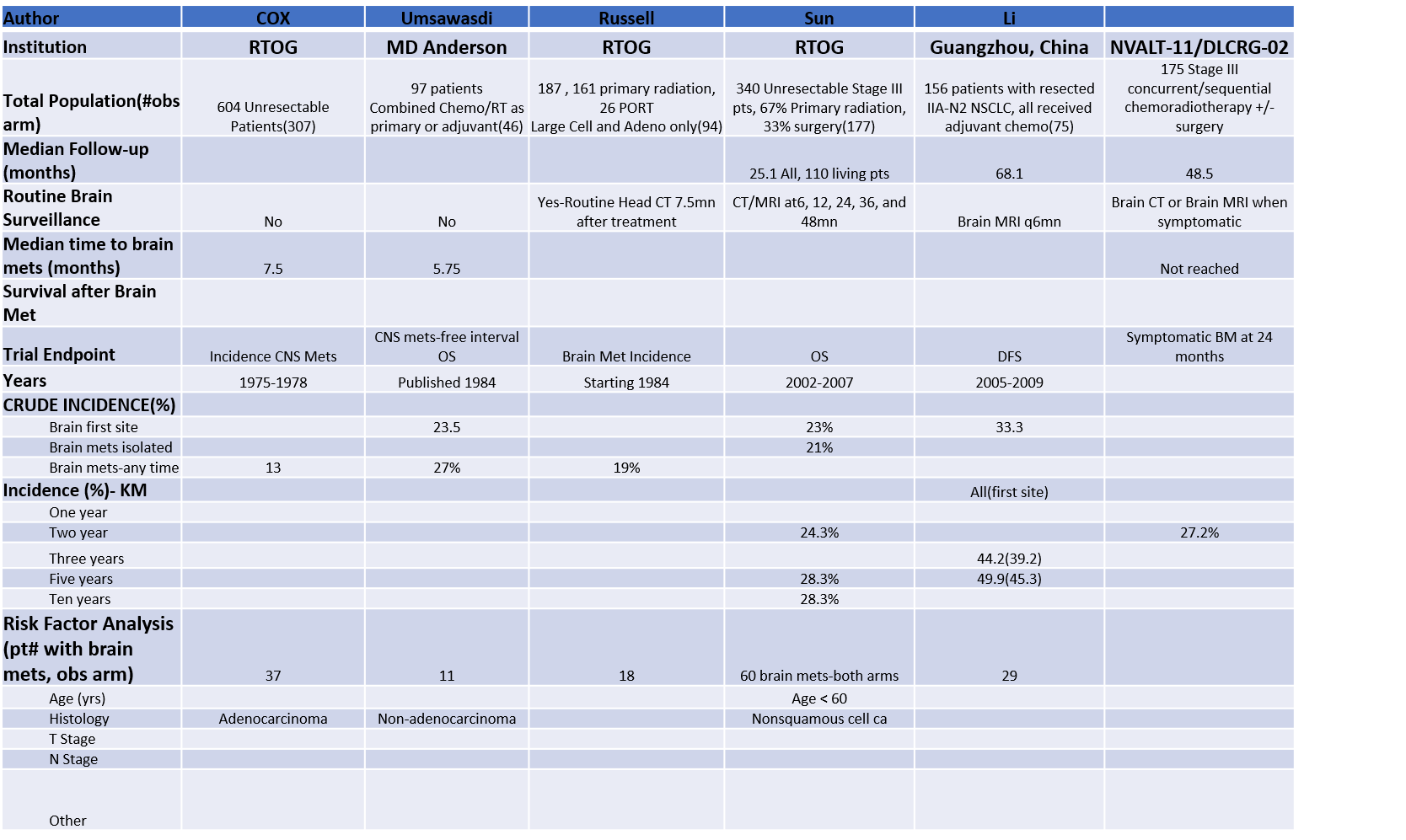

Supplement: Supplemental appendix [file mmc1.docx]
